# A-to-I RNA editing of BLCAP lost the inhibition to STAT3 activation in cervical cancer

**DOI:** 10.18632/oncotarget.17034

**Published:** 2017-04-11

**Authors:** Wen Chen, Wenrong He, Hongbing Cai, Bicheng Hu, Caishang Zheng, Xianliang Ke, Li Xie, Zhenhua Zheng, Xinxing Wu, Hanzhong Wang

**Affiliations:** ^1^ State Key Laboratory of Virology, Institute of Medical Virology, School of Basic Medical Sciences, Wuhan University, Wuhan 430071, China; ^2^ State Key Laboratory of Virology, Wuhan Institute of Virology, Chinese Academy of Sciences, Wuhan 430071, China; ^3^ Department of Gynaecology and Obstetrics, The First People's Hospital of Jingzhou, Yangtze University, Jingzhou 434000, China; ^4^ Department of Gynecologic Oncology, Zhongnan Hospital of Wuhan University, Wuhan 430071, China

**Keywords:** A-to-I RNA editing, BLCAP gene, cervical carcinogenesis, STAT3, YXXQ motif

## Abstract

Bladder cancer-associated protein (BLCAP) gene is a highly conserved gene with tumor-suppressor function in different carcinomas. It is also a novel ADAR-mediated editing substrate undergoes multiple A-to-I RNA editing events. Although the anti-tumorigenic role of BLCAP has been examined in preliminarily studies, the relationship between BLCAP function and A-to-I RNA editing in cervical carcinogenesis still require further exploration. Herein, we analyzed the coding sequence of *BLCAP* transcripts in 35 paired cervical cancer samples using high-throughput sequencing. Of note, editing levels of three novel editing sites were statistically different between cancerous and adjacent cervical tissues, and editing of these three sites was closely correlated. Moreover, two editing sites of BLCAP coding region were mapped-in the key YXXQ motif which can bind to SH2 domain of STAT3. Further studies revealed that BLCAP interacted with signal transducer and activator of transcription 3 (STAT3) and inhibited its phosphorylation, while A-to-I RNA editing of BLCAP lost the inhibition to STAT3 activation in cervical cancer cell lines. Our findings reveal that A-to-I RNA editing events alter the genetically coded amino acid in BLCAP YXXQ motif, which drive the progression of cervical carcinogenesis through regulating STAT3 signaling pathway.

## INTRODUCTION

Cervical cancer is the fourth most common cancer among female cancer patients, with an especially high prevalence in developing countries. It is also a deadly malignancy which caused 265,700 deaths worldwide in 2012 [1]. Although high-risk human papillomavirus (HPV) is well established as the major environmental risk factor for the occurrence of cervical cancer, the multiple processes of genetic and epigenetic alterations leading to the disease still need to be elucidated.

*BLCAP* is a highly conversed gene with two exons and an intron encoding a 10kDa protein, which was originally identified from invasive bladder carcinoma in 2002 [2]. We previously published data from *in vitro* and *in vivo* experiments indicated that *BLCAP* might be a potential anti-tumor gene in cervical cancer [3]. Other researchers reported that overexpression of BLCAP induced cell growth inhibition and apoptosis, suggested that BLCAP may function as a potential tumor suppressor in carcinogenesis through regulating cell proliferation and survival [4–6]. In addition, BLCAP represented a negative correlation with tumor progression by comparing its expression pattern in different grades and stages of bladder cancer and breast cancer, suggesting that it might be of value as a biomarker and providing a new perspective for the clinical application of this protein [7, 8].

Another interesting characteristic of *BLCAP* is that it is subject to adenosine to inosine (A-to-I) RNA editing. A-to-I RNA editing is an important post-transcription modification of RNA sequence, generating a diversity of RNA products different from the original DNA sequence. The adenosine deaminase acting on double-stranded RNA (ADAR) family of enzymes catalyze the conversion of adenosine (A) to inosine (I) in the double stranded RNA, and inosine is finally recognized as guanosine (G) in the process of mRNA translation [9]. As one of the representative substrates of A-to-I RNA editing identified in 2005 [10], BLCAP was reported to show an obvious difference in editing level between tumor and normal tissues, with a tumor-specific editing pattern in lung cancer, oral cavity cancer, brain cancer and urinary bladder cancer [11, 12]. In addition, the RNA edited *BLCAP* gene was found to stably promote cell proliferation, suggesting that the RNA over-editing of BLCAP may serve as a novel potential driver in advanced hepatocellular carcinoma [13].

Signal transducer and activator of transcription 3 (STAT3) is a transcription factor which regulates a variety of cellular physiological activities. In the canonical pathway, STAT3 is phosphorylated subsequent to Janus kinases (JAKs) activation by some key activators such as IL-6 family cytokines, and the activated STAT3 migrates to the nuclei to recognize the STAT3-specific DNA-binding elements. The main target genes of STAT3 include Bcl-2 family proteins, Cyclins and matrix metalloproteinases (MMPs), which are implicated in anti-apoptosis, pro-proliferation, induction of angiogenesis, promotion of metastasis and metastasis and evasion of anti-tumor immunity [14]. Constitutive activation of STAT3 is involved in a wide range of human cancers and associated with tumorigenesis, therefore, STAT3 is regarded as a promising target for cancer therapy [15, 16]. It has been reported that activated STAT3 was overexpressed in cervical cancer and could act as a predictor of poor prognosis, suggesting its potential role in progression of cervical carcinogenesis [17–19].

Although the function of BLCAP has been examined in preliminarily studies, how BLCAP plays an anti-oncogenic role and edited BLCAP functions as a cancer driver still require further exploration. Herein, we found that the editing levels of three novel editing sites were statistically different between cancerous and adjacent tissues by analyzing the coding sequence of *BLCAP* in 35 paired cervical cancer samples using high-throughput sequencing, and also observed that editing of these three sites was closely correlated. We predicted using Eukaryotic Linear Motif (ELM) that two editing sites of BLCAP transcript coding region were in the key YXXQ motif which can bind to SH2 domain of STAT3. Further studies revealed that BLCAP interacted with STAT3 and inhibited its phosphorylation, while A-to-I RNA edited BLCAP was unable to interact with STAT3 and failed to inhibit STAT3 phosphorylation as a result of editing the key adenosine in YXXQ motif of BLCAP. Our research delineate a new mechanism of BLCAP function as a tumor suppressor, and provide additional evidence that abnormal A-to-I RNA editing events alter the function of BLCAP by editing the critical sites and play a potential role in progression of cervical carcinogenesis.

## RESULTS

### The hyper-edited *BLCAP* is associated with cervical carcinogenesis

**Figure 1:**
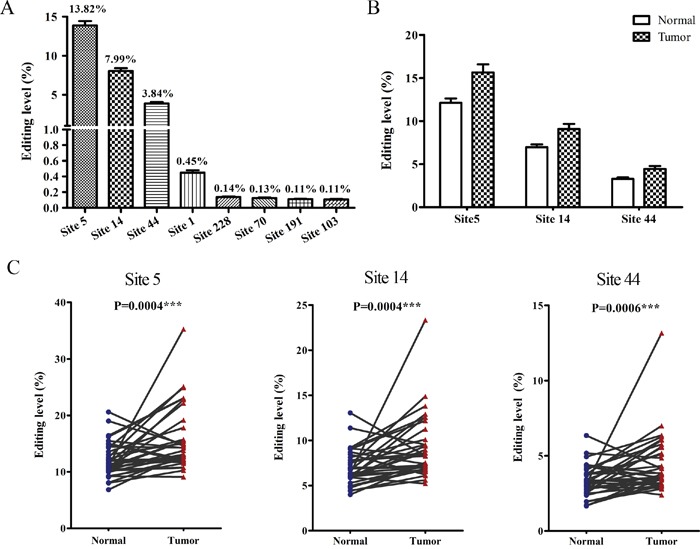
The hyper-edited *BLCAP* is associated with cervical carcinogenesis **(A)** Editing levels of eight edited adenosine sites in BLCAP coding sequence with an average editing level>0.1%. **(B** and **C)** Editing levels of three novel editing sites (site 5, site 14, site44) in 35 paired cervical cancer tissues (Tumor) and adjacent non-tumor tissues (Normal) (Related-samples Wilcoxon Signed Rank Test, ***P<0.001). Editing level in Figure 1 defined as the reads with indicated site edited to total reads in each sample.

The novel editing sites of *BLCAP* have been identified by bioinformatics and direct sequencing in several tissues and cell lines [20, 21]. Herein, we used high-throughput DNA sequencing instead of direct sequencing to provide a more thorough description of the A-to-I RNA edited sites of *BLCAP* in cervical cancer. We began with a total of 16,902,344 raw reads with Q20>71.35% and Q30>63.12% from 35 pairs of cervical cancer tissues and their adjacent non-tumor tissues (Supplementary Table 2), and obtained 4,874,614 perfectly matched reads after removing unqualified reads according to our strict criterion (see Materials and Methods). As we focused on the sites of A-to-I RNA editing which may cause the nonsynonymous codon changes and alter protein function, 39 adenosine sites in BLCAP coding sequence were analyzed. As shown in Figure 1A, site 5, site 14 and site 44 displayed the higher editing levels than others among 39 adenosine sites, with editing levels of 13.82%, 7.99% and 3.84% respectively. Except the first adenosine site (Site 1) which represented an editing level of 0.45%, editing levels of other adenosine sites were approximate or less than 0.1% (Figure 1A). Then we focused our attention on the three most highly edited sites to investigate the role of BLCAP A-to-I RNA editing in cervical carcinogenesis. Moderate elevations of editing at these three sites were noted in malignant tissues (1.29-, 1.30-, 1.35-fold increases respectively, Figure 1B), and editing levels of all the three sites showed significant difference between cervical tumor and their matched non-tumor tissues (Figure 1C). Collectively, these results suggest that the three novel hyper-edited sites in BLCAP coding sequence are closely associated with cervical carcinogenesis.

### Correlations among the three novel editing sites

**Figure 2:**
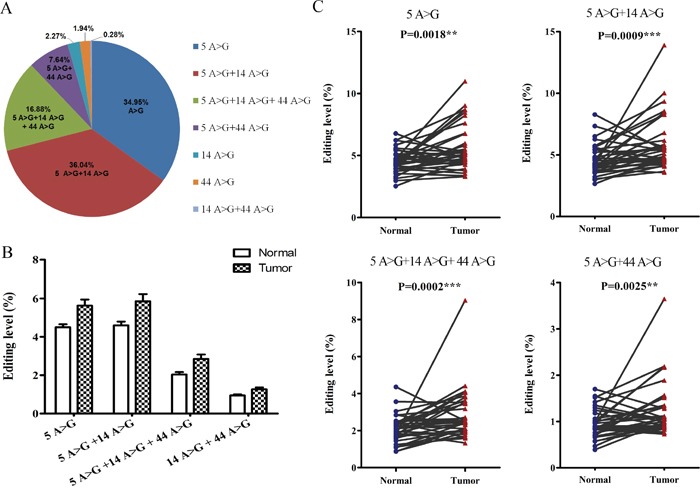
Correlations among the three novel editing sites **(A)** Percentage of seven cases with site 4 or/and site 14 or/and site 44 edited. **(B** and **C)** Editing levels of four hyper-edited cases between cervical cancer tissues (Tumor) and adjacent non-tumor tissues (Normal) (Related-samples Wilcoxon Signed Rank Test, **P<0.01, ***P<0.001). Editing level in Figure 2 defined as the reads of each case to total reads in each sample.

Although the RNA-edited *BLCAP* gene was reported serving as a novel potential driver in advanced HCC by *in vitro* and *in vivo* assays, the internal relationships among the editing sites have not yet been determined. In our previous study, we cloned PCR products from five pairs of cervical cancer tissues and sequenced multiple individual transcripts, then obtained 267 reads including 216 wild type reads and 51 mutant reads. Interestingly, we found that 16 reads showed site 5 A-to-G conversion, 15 reads exhibited site 5 and site14 A-to-G conversion, and 8 reads presented both site 5, site 14, site 44 A-to-G conversion among the 51 mutants (Supplementary Table 3). Thus, we hypothesized that editing events at the three novel editing sites might be connected. There were eight different types of transcripts when we focused on the three novel editing sites: three transcripts with single edits at site 5 (5 A>G), site 14 (14 A>G) or site 44 (44 A>G); three transcripts with double edits at both site 5 and site 14 (5 A>G + 14 A>G), site 5 and site 44 (5 A>G + 44 A>G), site 14 and site 44 (14 A>G + 44 A>G); one transcript with simultaneous edits at all three sites (5 A>G + 14 A>G + 44 A>G); and one transcript with no edited sites. To confirm the directly sequencing consequence, high-throughput sequencing database was classified in accordance with the eight cases. There were 692,100 reads containing the seven edited cases, making up 14.20% of the total reads (Supplementary Table 4). The percentage of four cases with site 5 edited (5 A>G, 5 A>G + 14 A>G, 5 A>G + 14 A>G + 44 A>G, 5 A>G + 44 A>G) was 34.95%, 36.04%, 16.88%, 7.64% respectively, accounting for the vast majority of the edited reads (the percentage added up to 95.51%, Figure 2A). As shown in Figure 2B and 2C, the editing level of the four cases was 1.25-, 1.28-, 1.34-, 1.40-fold higher in the cancerous tissues relative to the adjacent normal control (5.63% vs. 4.50%, 5.85% vs. 4.59%, 2.85% vs. 2.04%, 1.26% vs. 0.95% respectively) with statistically significant difference (P=0.0018, 0.0009, 0.0002, 0.0025, respectively). Taken together, these results show a correlation among the three novel editing sites in BLCAP coding sequence, and suggest that the editing pattern may be associated with pathogenesis of cervical cancer.

### ADAR1 plays an important role in BLCAP A-to-I RNA editing

**Figure 3:**
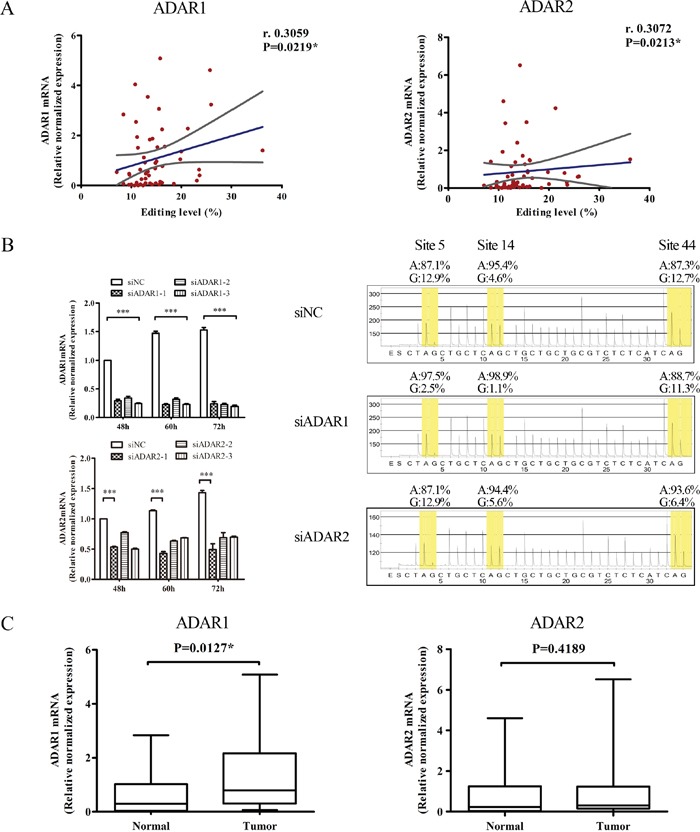
ADAR1 plays an important role in BLCAP A-to-I RNA editing **(A)** Correlations between BLCAP editing levels and the relative normalized mRNA levels of ADAR1 (left), ADAR2 (right) of 28 paired cervical specimens (56 individual samples), respectively. Blue lines represented linear regression, and grey lines represented the 95% confidence interval (Spearman correlation coefficients test, *P<0.05). Editing level in Figure 3 defined as the edited reads to total reads in each sample. **(B)** Three types of siADAR1, siADAR2 and siNC were transfected into HeLa cells and harvested after 48-72 hours. Cells were conducted with realtime-PCR and pyrosequencing assays. In the sequencing figures, yellow part represents the frequencies of original adenosine (A) and edited guanosine (G). Bases were skipped when there was no signal and overlapped when adjacent bases were the same. **(C)** Relatively expression of ADAR1 and ADAR2 between cervical cancer tissues (Tumor) and matched non-tumor tissues (Normal). The data are presented as box plots with media (horizontal line), 25-75% (box) and 5-95% (error bar) percentiles for each group (Related-samples Wilcoxon Signed Rank Test, *P<0.05).

Three enzymes participating in the A-to-I RNA editing are ADAR1, ADAR2 and ADAR3. Since ADAR3 had no documented deaminase activity and specifically expressed in the nervous system [22], we focused on ADAR1 and ADAR2 to study their role in BLCAP A-to-I RNA editing as well as cervical carcinogenesis. The mRNAs of ADAR1 and ADAR2 were both detectable in 28 out of 35 matched cervical samples by realtime-PCR assay. Among 28 paired cervical samples, both ADAR1 and ADAR2 showed a positive correlation between the relative normalized quantification value and the editing level of *BLCAP* (ADAR1: r=0.3059, P=0.0219; ADAR2: r=0.3072, P=0.0213; Figure 3A). To validate whether BLCAP A-to-I RNA editing was regulated by ADAR1 and ADAR2, we knockdown ADAR1 and ADAR2 in HeLa cells respectively and tested BLCAP editing level by pyrosequencing. As shown in Figure 3B, we first confirmed the knockdown efficiency of three corresponding siRNAs to silence human ADAR1 and ADAR2, and chosen siADAR1-3 and siADAR2-1 for the follow-up experiment. Pyrosequencing analysis of BLCAP mRNA-derived cDNA indicated that BLCAP editing levels of site 5, site 14 and site 44 were significantly decreased after ADAR1 knockdown, while only site 44 showed a reduced editing level in ADAR2 knockdown HeLa cells compared with the negative control (siNC transfected cells). In addition, 71.43% (20/28) cervical cancer specimens demonstrated higher expression of ADAR1 than their matched tumor specimens, and the percentage of cancerous specimen overexpression of ADAR2 was 64.29% (18/28). Comparing the ΔCt value of ADAR1 with ADAR2, there was a 2.39-cycle decrease in ADAR1 and a 1.13-cycle decrease in ADAR2, indicating that ADAR1 increased 5.2 fold while ADAR2 increased only 2.18 fold during cervical cancer development (Supplementary Figure 1). By comparing the relative expression of ADAR1 and ADAR2, only ADAR1 was significantly up-regulated (P=0.0127), while ADAR2 showed no significant difference (P=0.4189) in tumor samples compared to matched normal control tissues (Figure 3C). These date suggest that ADAR1 plays a more important role than ADAR2 in BLCAP A-to-I RNA editing and the progression of cervical cancer.

### BLCAP interacts with STAT3

**Figure 4:**
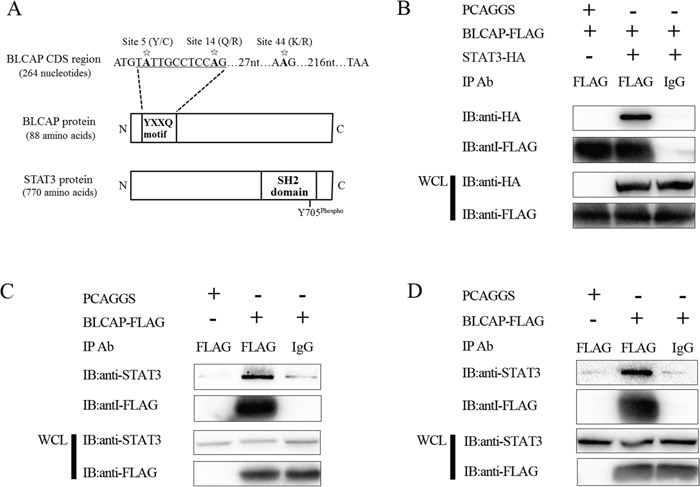
BLCAP interacts with STAT3 **(A)** Schematic representation of YXXQ motif of BLCAP and SH2 domain of STAT3. Asterisks represent the three novel A-to-I RNA editing sites in BLCAP coding sequence. **(B)** 293T cells were co-transfected with 3×FLAG-BLCAP and HA-STAT3 or 3×FLAG-BLCAP and empty vector PCAGGS. 24h later, the cells were harvested and lysed. Then the lysates were immunoprecipitated with FLAG antibody and normal mouse IgG. Immunoblot analysis was performed with anti-HA and anti-FLAG antibodies to detect the precipitated protein and the expression of transfected protein (WCL). **(C** and **D)** 293T cells **(C)** and HeLa cells **(D)** were transfected with 3×FLAG-BLCAP or PCAGGS respectively. The cells were harvested and lysed after 24h, and the lysates were immunoprecipitated with FLAG antibody and normal mouse IgG. Immunoblot analysis was performed with anti-STAT3 and anti-FLAG antibodies to detect the precipitated protein and the expression of transfected protein (WCL).

To explore the influence of BLCAP editing on the development of cervical cancer, we used ELM software from the Eukaryotic Linear Motif server at http://elm.eu.org/ to predict the potential proteins that could interact with BLCAP. Then, result that BLCAP YXXQ motif (where X is any amino acid) which contains the 5 and 14 editing sites could bind to signal transducer and activator of transcription 3 (STAT3) arouse our attention (Figure 4A). To date, there were previously reports that gp130 [23] and STAP-2 [24] which contain YXXQ motif are able to interact with STAT3 through binding to the SH2 domain and regulate JAK-STAT signaling pathway. To confirm whether BLCAP interacts with STAT3, 3×FLAG-BLCAP and HA-STAT3 were constructed and transfected into 293T and HeLa cells to perform Co-IP assays. First, we co-transfected the two plasmids into 293T cells, immunoprecipitated the cells lysates with an anti-FLAG antibody and then immunoblotted with anti-HA antibody. Results showed that BLCAP could interact with STAT3 in 293T cells (Figure 4B). To confirm the result under more physiological conditions, we tested the interactions between exogenous BLCAP and endogenous STAT3 in 293T and HeLa cells. As Figure 4C and 4D, STAT3 was present in immunoprecipitates brought down with anti-FLAG antibody, whereas no STAT3 co-precipitated when normal mouse IgG was used as a control antibody. Taken together, these results indicate that BLCAP is able to interact with STAT3.

### BLCAP inhibits IL-6 induced STAT3 activation

**Figure 5:**
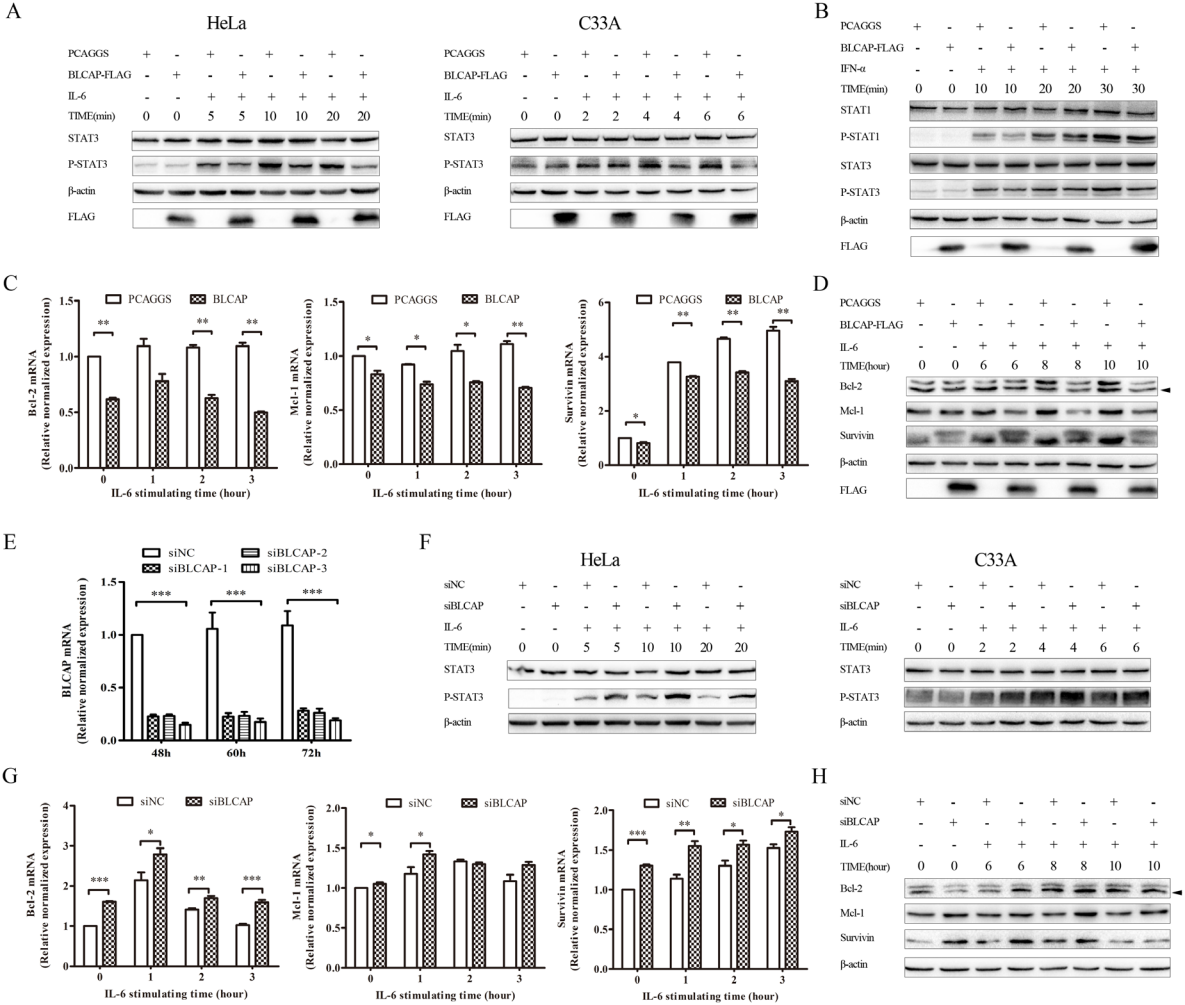
BLCAP inhibited IL-6 induced STAT3 activation **(A)** Empty PCAGGS vector or 3×FLAG-BLCAP plasmid was transfected into HeLa cells (left) and C33A cells (right) respectively. After 24h post-transfection, cells were treated with human IL-6 (10ng/ml for HeLa cells and 100ng/ml for C33A cells) and harvested at the indicated time. **(B)** PCAGGS or 3×FLAG-BLCAP was transfected into HeLa cells for 24 hours, cells were stimulated with human IFN-α (30ng/ml) for various lengths of time before harvested. **(C** and **D)** HeLa cells were transfected with PCAGGS and 3×FLAG-BLCAP for 24 hours, and treated with IL-6 (10ng/ml) for the indicated time before lysed. **(E)** SiNC and three types of siBLCAP were transfected into HeLa cells and harvested after 48-72 hours. **(F)** siNC or siBLCAP (siBLCACP-3) was transfected into HeLa (left) and C33A (right) cells for 72 hours. Cells were treated with IL-6 (10ng/ml for HeLa cells and 100ng/ml for C33A cells) for various length of time before lysed. **(G** and **H)** siNC or siBLCAP (siBLCACP-3) was transfected into HeLa cells for 72 hours. Cells were treated with IL-6 (10ng/ml) and harvested at the indicated time. All the harvested cells mentioned above were lysed and detected by realtime-PCR assay with different specific primers or Western blot analysis with the corresponding antibodies. β-actin was used as a loading control. The asterisk showed specific band. Data were shown by analysis of unpaired Student's t test, *P<0.05, **P<0.01, ***P<0.001.

To determine the function of interactions between BLCAP and STAT3, we examined the phosphorylated STAT3 (p-STAT3) as a prerequisite for STAT3 biological activity. As shown in Figure 5A, BLCAP inhibited IL-6 induced STAT3 tyrosine phosphorylation in HeLa cells, with rapid inhibition occurring as quickly as 10 minutes after exposure. We obtained the similar result in another cervical cancer cell line C33A cells. Since there is a high degree of homology between STAT1 and STAT3 [25], we examined the phosphorylation of STAT1 to determine if BLCAP had the same inhibition effect on STAT1. Both p-STAT1 and p-STAT3 showed a strongest expression after IFN-α stimulated for 30 minutes. When compared the relative expression of pSTAT1 and pSTAT3 (pSTAT/STAT) at 30 minutes by quantifying the bands with ImageLab software, pSTAT1 decreased in BLCAP transfected HeLa cells. However, the inhibition of BLCAP to STAT1 is not as significant as STAT3 (Figure 5B). We also tested the downstream target genes of STAT3, including Bcl-2, Mcl-1 and survivin at both mRNA and protein levels. As shown in Figure 5C and 5D, BLCAP inhibited the expression of Bcl-2, Mcl-1 and survivin with persistent IL-6 stimulation in accordance with the result of p-STAT3 inhibition.

To further confirm the involvement of BLCAP in regulating STAT3, we examined the phosphorylation of STAT3 by BLCAP knockdown. We first confirmed the knockdown efficiency and specificity of three corresponding siRNAs to silence human BLCAP at mRNA level, and siBLCAP-3 was chosen for the follow-up experiment (Figure 5E). BLCAP knockdown in both Hela and C33A cells resulted in enhanced phosphorylation of STAT3 in the presence of IL-6 (Figure 5F). Similarly, BLCAP knockdown increased the mRNA (Figure 5G) and protein (Figure 5H) level of Bcl-2, Mcl-1 and survivin, in accordance with the increase of p-STAT3 by BLCAP knockdown. Taken together, these data strongly suggest that BLCAP inhibits IL-6 induced STAT3 activation.

### Loss of STAT3 inhibition by edited BLCAP after YXXQ motif mutation

**Figure 6:**
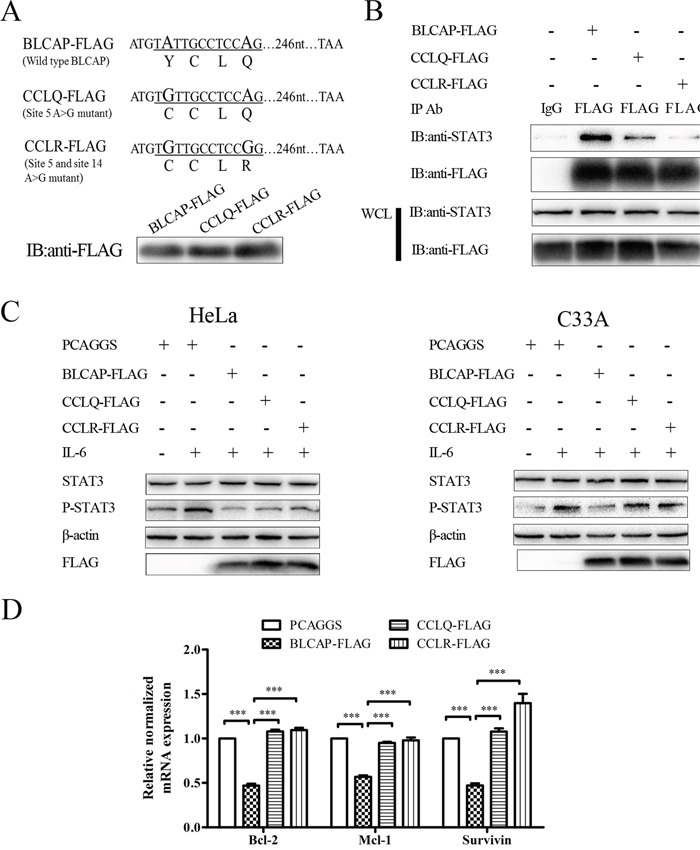
Loss of STAT3 inhibition by edited BLCAP after YXXQ motif mutation **(A)** The coding sequence of wild type BLCAP and two mutants. A>G substitutions (A-to-I RNA editing) in DNA sequence were oversized. Letters under the line represented the amino acid translated by DNA sequence underlined. Three types of BLCAP recombinant plasmids were transfected into HeLa cells and harvested after 24 hours, then Western blot was conducted with anti-FLAG antibody. **(B)** The wild type BLCAP and two mutants were transfected into HeLa cells respectively, followed by immunoprecipitation with an anti-FLAG antibody and immunoblot analysis with the indicated antibodies. **(C)** PCAGGS and three plasmids of BLCAP were transfected into HeLa (left) and C33A (right) cells for 24 hours. HeLa cells were harvested after IL-6 (10ng/ml) stimulated for 10 minutes and C33A cells were lysed after IL-6 (100ng/ml) stimulated for 4 minutes. The whole-cell lysates were processed for Western blot with the indicated antibodies. β-actin was used as a loading control. **(D)** PCAGGS and three types of BLCAP plasmids were transfected into HeLa cells for 24 hours to perform realtime-PCR assay. Data were shown by analysis of unpaired Student's t test, ***P<0.001.

Given that A-to-I RNA editing substitutes two important amino acids in BLCAP YXXQ motif, we constructed two edited plasmids to further explore the biological significance of BLCAP editing. One plasmid substituted A>G at site 5 (named CCLQ-FLAG) and the other changed A>G at both site 5 and site 14 (named CCLR-FLAG), and both two plasmids expressed the same molecular weight protein as wild type BLCAP (Figure 6A). We first explored the interaction of these two mutants with STAT3. As shown in Figure 6B, CCLQ-FLAG presented a notably weaker interaction compared with the wild type BLCAP, while CCLR-FLAG showed almost complete loss of STAT3 interaction. In agreement with the result of the immunoprecipitation assay, the phosphorylation of STAT3 increased in CCLQ-FLAG and CCLR-FLAG transfected HeLa cells as well as C33A cells compared with the wild type BLCAP transfected cells, indicating that the two mutants lost their ability to inhibit STAT3 (Figure 6C). As for the downstream genes, CCLQ-FLAG and CCLR-FLAG lost their ability to inhibit Bcl-2, Mcl-1 and survivin compared with wild type BLCAP (Figure 6D). These results collectively suggest that A-to-I RNA editing reverse BLCAP inhibition ability to STAT3 by editing the key adenosine in YXXQ motif.

## DISCUSSION

*BLCAP* was first identified as an ADAR-mediated edited gene through genomics and expressed sequence analyses [10]. So far, studies regarding the editing of *BLCAP* are mainly focused on the major aspects of the association with ADARs and the correlation with various diseases. The editing level of *BLCAP* differs greatly among cancer types. Angela Gallo's team found that editing of the *BLCAP* transcript was downregulated in astrocytomas, bladder cancer and colorectal cancer [21], while other groups reported a general elevation of editing levels in cancerous tissues such as liver, brain, oral cavity and lung [11, 13]. In addition, although the level of BLCAP hyper-editing showed a statistically significant difference between normal and tumoral urinary bladder cancer tissues, RNA editing did not participate in the evolution of urinary bladder cancer as observed through the analysis of nine editing substrates [12]. Studies mentioned above suggest that the editing level of *BLCAP* varies greatly depending on the tissue analyzed, and BLCAP A-to-I RNA editing may play a complex role in the development of different cancer types. Herein, we explored the editing level of *BLCAP* in cervical cancer by sequencing 35 paired specimens using high-throughput sequencing, and found that the editing level of site 5, site 14 and site 44 in cervical cancer tissues was significantly higher compared with their matched normal tissue, indicated that BLCAP hyper-editing might play an important role in the evolution of cervical cancer. Besides the three novel hyper-edited sites, other adenosine sites in BLCAP coding sequence showed low editing levels of approximate 0.1%. However, the editing level of site 1, site 103, site 122 and site 178 were statistically different between the cervical tumor and adjacent tissues, and site 103 showed a significant difference with P = 0.003 (Supplementary Figure 2). This result provide a clue that there may be other A-to-I RNA editing sites in BLCAP coding sequence associated with the function of BLCAP as well as the development of cervical cancer.

In addition, although ADAR1-mediated editing characteristics of *BLCAP* has been reported in ADAR knockdown cells lines [26], ADAR knockout mice [27] and clinical HCC samples [13], the relationship between ADARs with *BLCAP* in cervical tissues remains little understood. In this present investigation, we detected the mRNA expression of ADAR1 and ADAR2 in paired-cervical tissues by realtime-PCR and analyzed their correlation with BLCAP editing as well as cervical cancer. Results indicated that both ADAR1 and ADAR2 were involved in BLCAP editing, while ADAR1 played a more important role in BLCAP editing as well as cervical cancer. As ADAR1 was reported to function as an oncogene and ADAR2 function as a tumor-suppressive gene, we hypothesize that ADAR1 rather than ADAR2 dominate the conversion of *BLCAP* from a wild-type tumor-suppressor gene to an edited-type loss-of-function gene and finally promote the occurrence and development of cervical cancer. More experimentation will be required in order to verity this hypothesis.

A-to-I RNA editing affects numerous adenosine sites in the human transcriptome, including non-coding regions, coding regions, micro-RNAs. Several studies reveal that specific substitutions in the coding region can almost reverse the function of the edited substrates. There is one well-studied example of the glutamate receptor subunit, Glu-B, which requires editing in the Arg6807 by ADAR2 to carry out its physical function, while the unedited version drives the development of glioblastoma [28]. In contrast, AZIN1, another A-to-I RNA editing substrate which showed a gain-of-function event driving HCC pathogenesis when edited by ADAR1 at codon 376, presented the opposite editing function from Glu-B [29]. In this study, we verified that wild type BLCAP inhibited IL-6 induced STAT3 activation, while the two edited forms of BLCAP (CCLQ-FLAG and CCLR-FLAG) partly or almost failed to inhibit STAT3 phosphorylation. This result indicated that A-to-I RNA editing drove anti-tumorigenic BLCAP to a loss-of-function one which might facilitate the cervical cancer initiating and progressing events. Taken together, these researches suggest that the effect of A-to-I RNA editing on its target gene is gene-specific.

To our knowledge, this study is the first to propose the YXXQ motif in BLCAP. The YXXQ motif has been reported in several membrane transducing receptors which activate JAK-STAT signaling. In the gp130 YXXQ-mediated JAK-STAT pathway, STAT proteins are recruited to the phosphorylated YXXQ motif and then phosphorylated by JAKs upon IL-6 stimulation [23, 30]. In addition, Signal-transducing adaptor protein-2 (STAP-2) was reported to interact with the SH2 domain of STAT3 and activate STAT3 phosphorylation rely on its YXXQ motif [31]. All these studies suggest that the YXXQ motif function as an important activator to STAT3 activation. However, our study found that the YXXQ motif in BLCAP could interact with STAT3 but inhibit the IL-6 induced STAT3 phosphorylation as well as the downstream target genes of STAT3. This opposite result from the aforementioned studies thus indicates that YXXQ motif may exert different effect in regulating the activation of STAT3 depending on the specific protein.

Constitutively activated STAT3 plays an important role in oncogenic signaling and high expression level predicted poor prognosis, suggesting that inhibition of STAT3 may be an effective target for cancer therapy [15, 16]. Our work delineate the underlying molecular mechanisms of BLCAP-mediated inhibition of STAT3 in cervical cancer, and we hypothesis that BLCAP may act as a new potential therapeutic target for various types of STAT3-activated carcinomas. Thus, further investigations are required.

## MATERIALS AND METHODS

### Patient samples of cervical cancer tissues and adjacent normal tissues

Human cervical cancer and matched adjacent noncancerous specimens were collected from the Zhongnan Hospital of Wuhan University. This study was conducted in full compliance with the ethical principles of the Declaration of Helsinki and principles of good clinical practices, with approvals from the Ethics Committee of Zhongnan Hospital affiliated to Wuhan University. Written informed consents were obtained from all participants.

### Cells culture and treatments

The human embryonic kidney 293T cells and cervical cancer cell line HeLa cells (both from ATCC, Rockville, MD, USA) were cultured in DMEM (Gibco, Grand Island, NY, USA), C33A cells (ATCC) was cultured in MEM (Gibco). All media were supplemented with 10% heat-inactivated FBS (Gibco). The cells were maintained in a humidified incubator with 5% CO_2_ and 95% air at 37°C. Plasmids were transfected into 293T cells using the ProFection Mammalian Transfection System (Promega, Madison, WI, USA), and transfected into HeLa and C33A cells using Lipofectamine™2000 (Invitrogen, Carlsbad, CA, USA) following manufacturers' specifications. Silencing RNAs (siRNAs) and negative control (siNC) sequences (Supplementary Table 1) were chemically synthesized by the RiboBio Company (Guangzhou, China) and transfected into HeLa cells using Lipofectamine™3000 (Invitrogen) according to the manufacturer's instructions. In some cases, transfected cells were exposed to IL-6 for the indicated time before lysis.

### Plasmids, antibodies and cytokines

Human full-length STAT3 cDNA was amplified from a whole human cDNA library and was cloned into PCAGGS-HA with specific primers (Supplementary Table 1), The recombinant pEF-BLCAP-3×FLAG plasmid was established and stored in our laboratory. Two edited versions of BLCAP were amplified by PCR with site specific primers (Supplementary Table 1) from the plasmid pEF-BLCAP-3×FLAG. All constructs were confirmed by DNA sequencing. The antibodies used in this study included: mouse anti-FLAG mAb, rabbit anti-HA mAb (both from Sigma-Aldrich, St. Louis, MO, USA); rabbit anti-human mAbs against STAT3, p-STAT3 (both from Cell Signaling Technology, Danvers, MA, USA); mouse anti-β-actin, rabbit anti-human mAbs against STAT1, p-STAT1, Bcl-2, Mcl-1 and survivin (both from Ruiying Biological, Suzhou, China). Mouse control IgGs was purchased from Santa Cruz Biotechnology (CA, USA). Human IL-6 was purchased from Proteintech (Proteintech Group, Wuhan, China).

### RNA extraction and PCR amplification

Total RNA was extracted from cervical tissue samples or cells using TRIzol Reagent (Life Technologies, Rockville, MD, USA), according to the manufacturer's standard protocol. First-stand cDNA was generated from total RNA (1 μg) for each sample using a PrimeScript™ RT reagent kit (Takara, Dalian, China). Each cDNA samples was amplified with a set of primers targeting the 5′-UTR, coding region and 3′-UTR sequence, and a seven-base barcode sequence was designed to differentiate between samples (Supplementary Table 1). PCR was initiated in a 50 μl reaction volume using KOD-Plus-Neo (Toyobo, Osaka, Japan), and after extraction from a 2% agarose gel, amplicons were purified using an E.Z.N.A.™ Gel Extraction Kit (Omega Bio-tek, Georgia, USA) after extraction from 2% agarose gel.

### DNA library preparation and Illumina Miseq sequencing

DNA library preparation was performed using TruSeq DNA HT Sample Prep Kits (Illumina, San Diego, CA, USA). Briefly, the purified amplicons were performed with end repair and 3′ ends adenylate, then adapters were added by ligation and enrichment with a low-cycle according to the manufacturer's protocol. Then the purified DNA library products were evaluated using the Agilent 2200 TapeStation (Agilent Technologies, Wilmington, DE, USA) and Qubit 2.0 (Life Technologies) and pooled in equimolar ratios. The sequencing reaction was conducted on an Illumina Miseq PE300 platform (2×300 bp) in RiboBio Company, according to the standard procedures of Miseq reagent kits v3 (Illumina).

### Bioinformatics analysis

Fastq files were generated by bcl2fastq2.17 (Illumina). Raw fastq files were quality-filtered with FastQC (Illumina) to obtain clean reads with the following criteria: (1) Reads polluted by adapter sequences were removed; (2) reads with N rate more than 10% were removed; (3) reads which had more than 20% of bases with quality ≤Q20 were removed. To obtain perfectly matched reads, clean reads required further selection based on two additional criteria: (1) Reads were removed with more than five bases mismatched when merged with the last 50 bases of read1 and read2 by FLASH (version 1.2.6); (2) reads were removed with more than ten single nucleotide polymorphism sites detected by sequence alignment using BLAST (version 2.2.30). The subsequent statistical analysis and data visualization were performed using programming language R.

### Pyrosequencing

Pyrosequencing was performed using PyroMark Q96 Reagent (Qiagen, Duesseldorf, German). First, PT-PCR was performed with biotin labeled forward primer and reverse primer (Supplementary Table 1) to obtain the single stranded biotinylated PCR products. Second, we mixed the sepharose bead with PCR product for 5 minutes at room temperature and prepared the vacuum prep tool in vacuum prep workstation to capture the beads. After denaturing and washing, template mixture with sequencing primer was transferred to a PSQ 96 plate and heated to 80°C for 2 minutes. Sequencing reactions were performed with PyroMark Gold Q96 ID (Qiagen, Duesseldorf, German) and analysed with PyroMark Gold Q96 ID software (Qiagen, Duesseldorf, German) in Sangon Biotech (Shanghai, China).

### Real-time PCR analysis

Real-time PCR was conducted with a total volume of 20 μL iQ™SYBR Green Supermix (Bio-Rad, California, USA) containing cDNA product (2 μl) and specific primers (Supplementary Table 1) on a CFX Connect Real-time PCR Detection System from Bio-Rad. The amounts of target transcripts were calculated and normalized with housekeeping gene GAPDH in the tissues. Relative ADAR1 and ADAR2 expression levels in the tissues were calculated as 2^−ΔCT^ (ΔC_T_=C_T_^ADAR1/ADAR2^-C_T_^GAPDH^) and normalized to the average relative expression level in all non-tumor tissues, which was defined as 1.0. The relative ADAR1, ADAR2, BLCAP, Bcl-2, Mcl-1 and survivin expression levels in the cells were given as 2^−ΔCT^ (ΔC_T_=C_T_^BLCAP/Bcl-2/Mcl-1/Survivin^-C_T_^GAPDH^) and normalized to the expression in the corresponding control cells, which were defined as 1.0.

### Co-immunoprecipitation (Co-IP)

Transfected cells were harvested 24 h post-transfection and lysed with cell lysis buffer (Beyotime, Shanghai, China) supplemented with 1mM of the protease inhibitor PMSF. After centrifugation, the supernatant was incubated with 1 μg anti-FLAG antibody plus protein G magnetic beads (Invitrogen) for 20 minutes at room temperature. After six washes, the immunoprecipitates were eluted by boiling the beads for 10 minutes in SDS protein loading buffer and then western blot analysis was performed with corresponding antibodies. Mouse IgG was used as a negative control.

### Western blot analysis

Protein lysates were quantified, resolved on SDS-PAGE gels, transferred onto polyvinylidene difluoride membranes, and immunoblotted with primary antibodies overnight at 4°C, followed by incubation with a HRP-conjugated secondary antibody. Bands were visualized using the ChemiDoc™System (Bio-Rad).

### Statistical analysis

Results were reported as mean±SD. All statistical analyses were carried out using the SPSS standard version 20.0 software (IBM, Armonk, NY, USA). Related-samples Wilcoxon signed rank test, Spearman correlation coefficients test and unpaired Student's t test were used to compare the difference between any two preselected groups. P<0.05 was considered as statistically significant.

## SUPPLEMENTARY FIGURES AND TABLES




