# Supplementary material for: A-to-I RNA editing of BLCAP lost the inhibition to STAT3 activation in cervical cancer
Source: Oncotarget. 2017 Apr 11;8(24):39417–29. doi: 10.18632/oncotarget.17034 (PMC5503622; doi:10.18632/oncotarget.17034)
Supplement: Supplementary file 1 [file oncotarget-08-39417-s001.pdf]

A-to-I RNA editing of BLCAP lost the inhibition to STAT3 activation in cervical cancer

SUPPLEMENTARY FIGURES AND TABLES

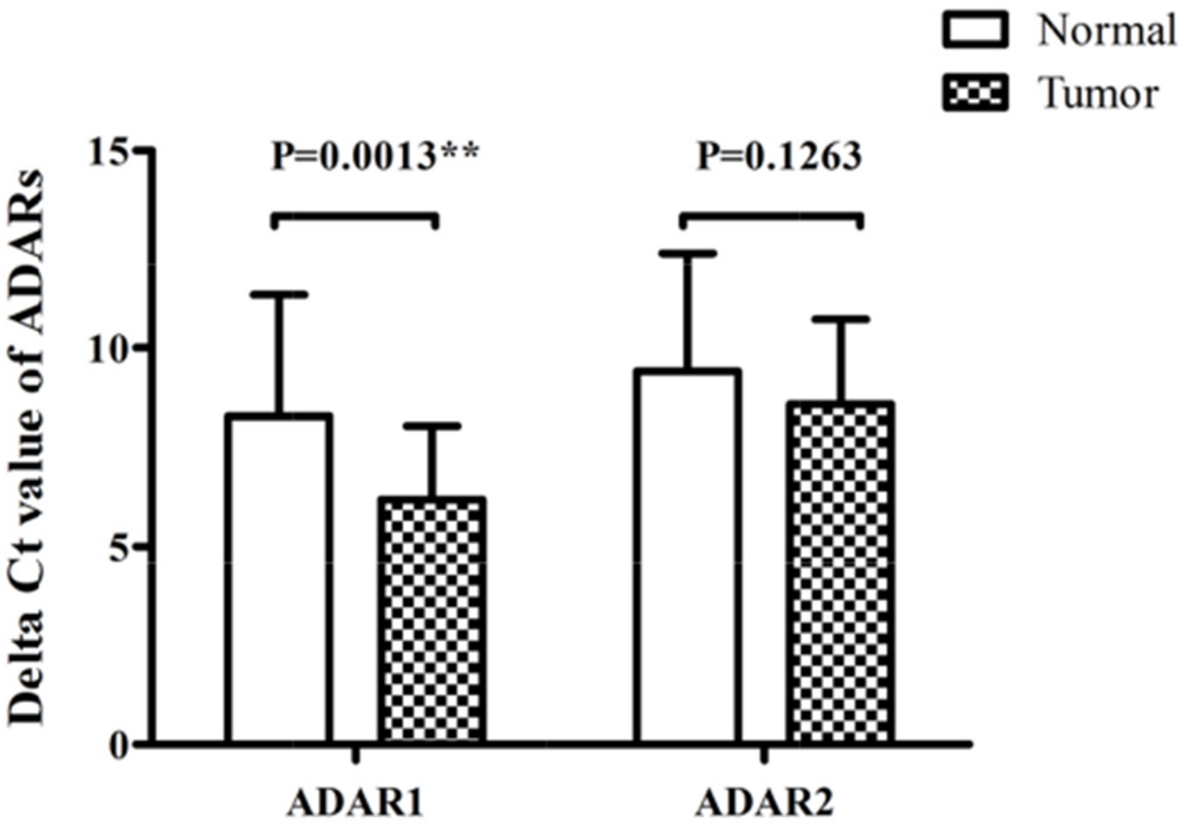

Supplementary Figure 1: Delta Ct value of ADAR1 and ADAR2 in 35 paired cervical cancer tissues. ΔCt value of ADAR1 and ADAR2 between cervical cancer (Tumor) and matched non-tumor (Normal) tissues were detected using real-time PCR (Related-samples Wilcoxon Signed Rank Test, **P<0.01)

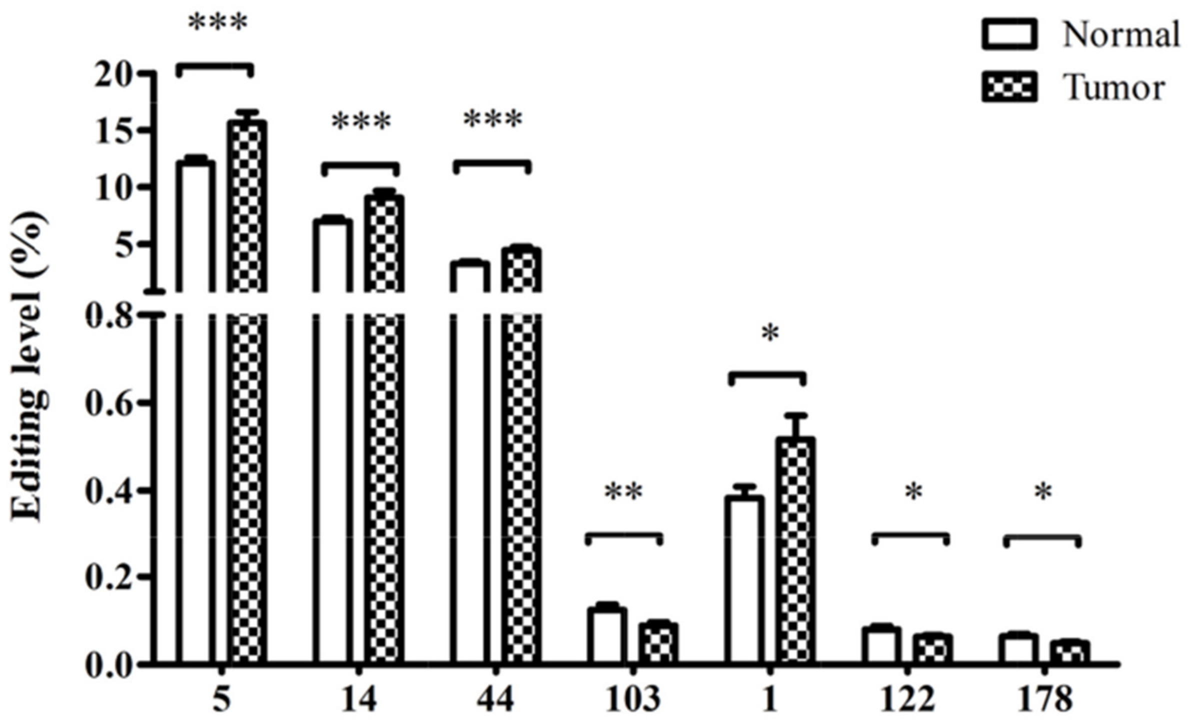

Supplementary Figure 2: Seven statistically different editing sites in BLCAP coding region. Seven editing sites among 39 adenosine sites located in BLCAP coding region were statistically different when compared the tumor tissues (Tumor) with their adjacent non–tumors (Normal) (Related-samples Wilcoxon Signed Rank Test, *P<0.05, **P<0.01, ***P<0.001)

Supplementary Table 1: SiRNA sequences and primer sequences used in this study

See Supplementary File 1

Supplementary Table 2: Quality and data filtering of 35 paired cervical samples

See Supplementary File 1

Supplementary Table 3: Direct sequencing of BLCAP in 5 paired cervical cancer tissues

	5 A>G	44 A>G	5 A>G +14 A>G	5 A>G+14 A>G +44 A>G	Other types	Total
n.clones	16	1	15	8	11	51
Percentage (%)	31.37	1.96	29.41	15.69	21.57	100

Five paired specimens were randomly selected from 35 paired cervical cancer tissues. After RNA extraction and PCR amplification with BLCAP specific primers, we cloned each of the purified PCR products into pGEM-T easy vector and sequenced the plasmids. A total of 267 reads were obtained from 5 paired samples, with 216 wild type reads and 51 mutant reads. Among the 51 mutant reads, 16 reads showed site 5 A-to-G conversion, 1 reads showed site 44-A to-G conversion, 15 reads showed site 5 and site14 A-to-G conversion, and 8 reads showed both site 5, site 14, site 44 A-to-G conversion. The percentage of the four cases was 31.37%, 1.96%, 29.41% and 15.69% respectively

Supplementary Table 4: Numbers of reads classified into eight cases in high-throughput sequencing database

See Supplementary File 1
