# Supplementary material for: A-to-I RNA editing of BLCAP lost the inhibition to STAT3 activation in cervical cancer
Source: Oncotarget. 2017 Apr 11;8(24):39417–29. doi: 10.18632/oncotarget.17034 (PMC5503622; doi:10.18632/oncotarget.17034)
Supplement: Supplementary file 2 [file oncotarget-08-39417-s002.docx]

**Supplementary Table 1. SiRNA sequences and primer sequences used in this study.**

| **Method** | **Target** | **Number** | **Sequence** |  |
| --- | --- | --- | --- | --- |
| siRNA | BLCAP | 1 | CCCTGTTCCTTATCTGCTA |  |
|  | BLCAP | 2 | GCACAATTTGTGCCTTGGT |  |
|  | BLCAP | 3 | CCATGTTCATGGGCTTCTA |  |
| siRNA | ADAR1 | 1 | GCATCTGACCCGTGCTATT |  |
|  | ADAR1 | 2 | CATCAAATGCCTCAAATAA |  |
|  | ADAR1 | 3 | GCTTCAACACTCTGACTAA |  |
| siRNA | ADAR2 | 1 | CAGGCACAGATGTTAAAGA |  |
|  | ADAR2 | 2 | CCGCTATTGAGGTCATCAA |  |
|  | ADAR2 | 3 | GAAGGAGAATGTCCAGTTT |  |
| **Method** | **Target** | **Number** | **Forward primer** | **Reverse primer** |
| Genomic PCR | HA-STAT3 |  | 5’-GCGCTCGAGCCACCATGGCCCAATGGAATCAGCTAC-3’ | 5’-CCCGGGTTAAGCGTAATCTGGAACATCGTATGGGTACATGGGGGAGGTAGCGCAC-3’ |
| Mutant PCR | CCLQ-FLAG |  | 5’-CGGCTTAAGATGTGTTGCCTCCAGTG-3’ | 5’-ACGACGCGTGGTGCCCACAACGC-3’ |
|  | CCLR-FLAG |  | 5’-CGGCTTAAGATGTGTTGCCTCCGGTG-3’ | 5’-ACGACGCGTGGTGCCCACAACGC-3’ |
| Real-time PCR | ADAR1 |  | 5’-GCTTGGGAACAGGGAATCG-3’ | 5’-CTGTAGAGAAACCTGATGAAGCC-3’ |
|  | ADAR2 |  | 5’-CGCAGGTTTTAGCTGACGC-3’ | 5’-GCATCTTTAACATCTGTGCCTGT-3’ |
|  | BLCAP |  | 5’-CAGGAAAACCAAGGCACAAA-3’ | 5’-CAGGAAAACCAAGGCACAAA-3’ |
|  | Bcl-2 |  | 5’-ATCGCCCTGTGGATGACTGAGT-3’ | 5’-GCCAGGAGAAATCAAACAGAGGC-3’ |
|  | Mcl-1 |  | 5’-CCAAGAAAGCTGCATCGAACCAT-3’ | 5’-CAGCACATTCCTGATGCCACCT-3’ |
|  | Survivin |  | 5’-GTATTACAGGCGTAAGCCACCG-3’ | 5’-CCACTGAGAACGAGCCAGACTT-3’ |
| Pyrosequence (PCR) | BLCAP |  | 5’-CTTGGTGAAGGCCCTGC-3’ | 5’-TGAGCAGGTAGAAGCCCAT-3’ |
| Pyrosequence (Biotin labled) | BLCAP |  | 5’-CCGGCAGAGATCATG-3’ |  |
| High-thoughput sequence | BLCAP | cDNA_201 | 5’-CCCTACACGACGCTCTTCCGATCTNCACTCTACAGGATCCCTGCTGCCTTGG-3’ | 5’-GACTGGAGTTCCTTGGCACCCGAGAATTCCACTCCCCTCCCGTCTTCTGCT-3’ |
|  |  | cDNA_202 | 5’-CCCTACACGACGCTCTTCCGATCTNCTGAGACCAGGATCCCTGCTGCCTTGG-3’ | 5’-GACTGGAGTTCCTTGGCACCCGAGAATTCCACTCCCCTCCCGTCTTCTGCT-3’ |
|  |  | cDNA_203 | 5’-CCCTACACGACGCTCTTCCGATCTNTCATCTGCAGGATCCCTGCTGCCTTGG-3’ | 5’-GACTGGAGTTCCTTGGCACCCGAGAATTCCACTCCCCTCCCGTCTTCTGCT-3’ |
|  |  | cDNA_204 | 5’-CCCTACACGACGCTCTTCCGATCTNGTGCGATCAGGATCCCTGCTGCCTTGG-3’ | 5’-GACTGGAGTTCCTTGGCACCCGAGAATTCCACTCCCCTCCCGTCTTCTGCT-3’ |
|  |  | cDNA_205 | 5’-CCCTACACGACGCTCTTCCGATCTNATGTGTGCAGGATCCCTGCTGCCTTGG-3’ | 5’-GACTGGAGTTCCTTGGCACCCGAGAATTCCACTCCCCTCCCGTCTTCTGCT-3’ |
|  |  | cDNA_206 | 5’-CCCTACACGACGCTCTTCCGATCTNCATGTCGCAGGATCCCTGCTGCCTTGG-3’ | 5’-GACTGGAGTTCCTTGGCACCCGAGAATTCCACTCCCCTCCCGTCTTCTGCT-3’ |
|  |  | cDNA_207 | 5’-CCCTACACGACGCTCTTCCGATCTNTCGTATGCAGGATCCCTGCTGCCTTGG-3’ | 5’-GACTGGAGTTCCTTGGCACCCGAGAATTCCACTCCCCTCCCGTCTTCTGCT-3’ |
|  |  | cDNA_208 | 5’-CCCTACACGACGCTCTTCCGATCTNATCTAGTCAGGATCCCTGCTGCCTTGG-3’ | 5’-GACTGGAGTTCCTTGGCACCCGAGAATTCCACTCCCCTCCCGTCTTCTGCT-3’ |
|  |  | cDNA_209 | 5’-CCCTACACGACGCTCTTCCGATCTNTGCGTAGCAGGATCCCTGCTGCCTTGG-3’ | 5’-GACTGGAGTTCCTTGGCACCCGAGAATTCCACTCCCCTCCCGTCTTCTGCT-3’ |
|  |  | cDNA_210 | 5’-CCCTACACGACGCTCTTCCGATCTNCACAGCACAGGATCCCTGCTGCCTTGG-3’ | 5’-GACTGGAGTTCCTTGGCACCCGAGAATTCCACTCCCCTCCCGTCTTCTGCT-3’ |
|  |  | cDNA_211 | 5’-CCCTACACGACGCTCTTCCGATCTNTCACATCCAGGATCCCTGCTGCCTTGG-3’ | 5’-GACTGGAGTTCCTTGGCACCCGAGAATTCCACTCCCCTCCCGTCTTCTGCT-3’ |
|  |  | cDNA_212 | 5’-CCCTACACGACGCTCTTCCGATCTNCTCAGATCAGGATCCCTGCTGCCTTGG-3’ | 5’-GACTGGAGTTCCTTGGCACCCGAGAATTCCACTCCCCTCCCGTCTTCTGCT-3’ |
|  |  | cDNA_213 | 5’-CCCTACACGACGCTCTTCCGATCTNATAGTGACAGGATCCCTGCTGCCTTGG-3’ | 5’-GACTGGAGTTCCTTGGCACCCGAGAATTCCACTCCCCTCCCGTCTTCTGCT-3’ |
|  |  | cDNA_214 | 5’-CCCTACACGACGCTCTTCCGATCTNGTACATACAGGATCCCTGCTGCCTTGG-3’ | 5’-GACTGGAGTTCCTTGGCACCCGAGAATTCCACTCCCCTCCCGTCTTCTGCT-3’ |
|  |  | cDNA_215 | 5’-CCCTACACGACGCTCTTCCGATCTNGTATAGTCAGGATCCCTGCTGCCTTGG-3’ | 5’-GACTGGAGTTCCTTGGCACCCGAGAATTCCACTCCCCTCCCGTCTTCTGCT-3’ |
|  |  | cDNA_216 | 5’-CCCTACACGACGCTCTTCCGATCTNACATATACAGGATCCCTGCTGCCTTGG-3’ | 5’-GACTGGAGTTCCTTGGCACCCGAGAATTCCACTCCCCTCCCGTCTTCTGCT-3’ |
|  |  | cDNA_217 | 5’-CCCTACACGACGCTCTTCCGATCTNGTGCTCGCAGGATCCCTGCTGCCTTGG-3’ | 5’-GACTGGAGTTCCTTGGCACCCGAGAATTCCACTCCCCTCCCGTCTTCTGCT-3’ |
|  |  | cDNA_218 | 5’-CCCTACACGACGCTCTTCCGATCTNGATATCGCAGGATCCCTGCTGCCTTGG-3’ | 5’-GACTGGAGTTCCTTGGCACCCGAGAATTCCACTCCCCTCCCGTCTTCTGCT-3’ |
|  |  | cDNA_219 | 5’-CCCTACACGACGCTCTTCCGATCTNGTGTCTGCAGGATCCCTGCTGCCTTGG-3’ | 5’-GACTGGAGTTCCTTGGCACCCGAGAATTCCACTCCCCTCCCGTCTTCTGCT-3’ |
|  |  | cDNA_220 | 5’-CCCTACACGACGCTCTTCCGATCTNACTCGCTCAGGATCCCTGCTGCCTTGG-3’ | 5’-GACTGGAGTTCCTTGGCACCCGAGAATTCCACTCCCCTCCCGTCTTCTGCT-3’ |
|  |  | cDNA_221 | 5’-CCCTACACGACGCTCTTCCGATCTNGACTGTCCAGGATCCCTGCTGCCTTGG-3’ | 5’-GACTGGAGTTCCTTGGCACCCGAGAATTCCACTCCCCTCCCGTCTTCTGCT-3’ |
|  |  | cDNA_222 | 5’-CCCTACACGACGCTCTTCCGATCTNAGTCGCGCAGGATCCCTGCTGCCTTGG-3’ | 5’-GACTGGAGTTCCTTGGCACCCGAGAATTCCACTCCCCTCCCGTCTTCTGCT-3’ |
|  |  | cDNA_223 | 5’-CCCTACACGACGCTCTTCCGATCTNGCGTCGCCAGGATCCCTGCTGCCTTGG-3’ | 5’-GACTGGAGTTCCTTGGCACCCGAGAATTCCACTCCCCTCCCGTCTTCTGCT-3’ |
|  |  | cDNA_224 | 5’-CCCTACACGACGCTCTTCCGATCTNAGAGACACAGGATCCCTGCTGCCTTGG-3’ | 5’-GACTGGAGTTCCTTGGCACCCGAGAATTCCACTCCCCTCCCGTCTTCTGCT-3’ |
|  |  | cDNA_225 | 5’-CCCTACACGACGCTCTTCCGATCTNTGTGTCACAGGATCCCTGCTGCCTTGG-3’ | 5’-GACTGGAGTTCCTTGGCACCCGAGAATTCCACTCCCCTCCCGTCTTCTGCT-3’ |
|  |  | cDNA_226 | 5’-CCCTACACGACGCTCTTCCGATCTNGAGCATACAGGATCCCTGCTGCCTTGG-3’ | 5’-GACTGGAGTTCCTTGGCACCCGAGAATTCCACTCCCCTCCCGTCTTCTGCT-3’ |
|  |  | cDNA_227 | 5’-CCCTACACGACGCTCTTCCGATCTNGCATCGTCAGGATCCCTGCTGCCTTGG-3’ | 5’-GACTGGAGTTCCTTGGCACCCGAGAATTCCACTCCCCTCCCGTCTTCTGCT-3’ |
|  |  | cDNA_228 | 5’-CCCTACACGACGCTCTTCCGATCTNGCGTGTGCAGGATCCCTGCTGCCTTGG-3’ | 5’-GACTGGAGTTCCTTGGCACCCGAGAATTCCACTCCCCTCCCGTCTTCTGCT-3’ |
|  |  | cDNA_229 | 5’-CCCTACACGACGCTCTTCCGATCTNATATGTACAGGATCCCTGCTGCCTTGG-3’ | 5’-GACTGGAGTTCCTTGGCACCCGAGAATTCCACTCCCCTCCCGTCTTCTGCT-3’ |
|  |  | cDNA_230 | 5’-CCCTACACGACGCTCTTCCGATCTNAGTGCTGCAGGATCCCTGCTGCCTTGG-3’ | 5’-GACTGGAGTTCCTTGGCACCCGAGAATTCCACTCCCCTCCCGTCTTCTGCT-3’ |
|  |  | cDNA_231 | 5’-CCCTACACGACGCTCTTCCGATCTNAGCTCATCAGGATCCCTGCTGCCTTGG-3’ | 5’-GACTGGAGTTCCTTGGCACCCGAGAATTCCACTCCCCTCCCGTCTTCTGCT-3’ |
|  |  | cDNA_232 | 5’-CCCTACACGACGCTCTTCCGATCTNTGTGTATCAGGATCCCTGCTGCCTTGG-3’ | 5’-GACTGGAGTTCCTTGGCACCCGAGAATTCCACTCCCCTCCCGTCTTCTGCT-3’ |
|  |  | cDNA_233 | 5’-CCCTACACGACGCTCTTCCGATCTNGATATGACAGGATCCCTGCTGCCTTGG-3’ | 5’-GACTGGAGTTCCTTGGCACCCGAGAATTCCACTCCCCTCCCGTCTTCTGCT-3’ |
|  |  | cDNA_234 | 5’-CCCTACACGACGCTCTTCCGATCTNGTCTGACCAGGATCCCTGCTGCCTTGG-3’ | 5’-GACTGGAGTTCCTTGGCACCCGAGAATTCCACTCCCCTCCCGTCTTCTGCT-3’ |
|  |  | cDNA_235 | 5’-CCCTACACGACGCTCTTCCGATCTNTATCGAGCAGGATCCCTGCTGCCTTGG-3’ | 5’-GACTGGAGTTCCTTGGCACCCGAGAATTCCACTCCCCTCCCGTCTTCTGCT-3’ |
|  |  | cDNA_236 | 5’-CCCTACACGACGCTCTTCCGATCTNTGCTGCGCAGGATCCCTGCTGCCTTGG-3’ | 5’-GACTGGAGTTCCTTGGCACCCGAGAATTCCACTCCCCTCCCGTCTTCTGCT-3’ |
|  |  | cDNA_237 | 5’-CCCTACACGACGCTCTTCCGATCTNTAGTCGCCAGGATCCCTGCTGCCTTGG-3’ | 5’-GACTGGAGTTCCTTGGCACCCGAGAATTCCACTCCCCTCCCGTCTTCTGCT-3’ |
|  |  | cDNA_238 | 5’-CCCTACACGACGCTCTTCCGATCTNACGTGCTCAGGATCCCTGCTGCCTTGG-3’ | 5’-GACTGGAGTTCCTTGGCACCCGAGAATTCCACTCCCCTCCCGTCTTCTGCT-3’ |
|  |  | cDNA_239 | 5’-CCCTACACGACGCTCTTCCGATCTNAGACATACAGGATCCCTGCTGCCTTGG-3’ | 5’-GACTGGAGTTCCTTGGCACCCGAGAATTCCACTCCCCTCCCGTCTTCTGCT-3’ |
|  |  | cDNA_240 | 5’-CCCTACACGACGCTCTTCCGATCTNCTAGCACCAGGATCCCTGCTGCCTTGG-3’ | 5’-GACTGGAGTTCCTTGGCACCCGAGAATTCCACTCCCCTCCCGTCTTCTGCT-3’ |
|  |  | cDNA_241 | 5’-CCCTACACGACGCTCTTCCGATCTNCTGCACGCAGGATCCCTGCTGCCTTGG-3’ | 5’-GACTGGAGTTCCTTGGCACCCGAGAATTCCACTCCCCTCCCGTCTTCTGCT-3’ |
|  |  | cDNA_242 | 5’-CCCTACACGACGCTCTTCCGATCTNCGCGATCCAGGATCCCTGCTGCCTTGG-3’ | 5’-GACTGGAGTTCCTTGGCACCCGAGAATTCCACTCCCCTCCCGTCTTCTGCT-3’ |
|  |  | cDNA_243 | 5’-CCCTACACGACGCTCTTCCGATCTNGCGACAGCAGGATCCCTGCTGCCTTGG-3’ | 5’-GACTGGAGTTCCTTGGCACCCGAGAATTCCACTCCCCTCCCGTCTTCTGCT-3’ |
|  |  | cDNA_244 | 5’-CCCTACACGACGCTCTTCCGATCTNGTGCTACCAGGATCCCTGCTGCCTTGG-3’ | 5’-GACTGGAGTTCCTTGGCACCCGAGAATTCCACTCCCCTCCCGTCTTCTGCT-3’ |
|  |  | cDNA_245 | 5’-CCCTACACGACGCTCTTCCGATCTNATAGCTACAGGATCCCTGCTGCCTTGG-3’ | 5’-GACTGGAGTTCCTTGGCACCCGAGAATTCCACTCCCCTCCCGTCTTCTGCT-3’ |
|  |  | cDNA_246 | 5’-CCCTACACGACGCTCTTCCGATCTNATGTCGTCAGGATCCCTGCTGCCTTGG-3’ | 5’-GACTGGAGTTCCTTGGCACCCGAGAATTCCACTCCCCTCCCGTCTTCTGCT-3’ |
|  |  | cDNA_247 | 5’-CCCTACACGACGCTCTTCCGATCTNGCGATCGCAGGATCCCTGCTGCCTTGG-3’ | 5’-GACTGGAGTTCCTTGGCACCCGAGAATTCCACTCCCCTCCCGTCTTCTGCT-3’ |
|  |  | cDNA_248 | 5’-CCCTACACGACGCTCTTCCGATCTNGCGACTACAGGATCCCTGCTGCCTTGG-3’ | 5’-GACTGGAGTTCCTTGGCACCCGAGAATTCCACTCCCCTCCCGTCTTCTGCT-3’ |
|  |  | cDNA_249 | 5’-CCCTACACGACGCTCTTCCGATCTNCGAGTACCAGGATCCCTGCTGCCTTGG-3’ | 5’-GACTGGAGTTCCTTGGCACCCGAGAATTCCACTCCCCTCCCGTCTTCTGCT-3’ |
|  |  | cDNA_250 | 5’-CCCTACACGACGCTCTTCCGATCTNCACACTGCAGGATCCCTGCTGCCTTGG-3’ | 5’-GACTGGAGTTCCTTGGCACCCGAGAATTCCACTCCCCTCCCGTCTTCTGCT-3’ |
|  |  | cDNA_251 | 5’-CCCTACACGACGCTCTTCCGATCTNGACGTAGCAGGATCCCTGCTGCCTTGG-3’ | 5’-GACTGGAGTTCCTTGGCACCCGAGAATTCCACTCCCCTCCCGTCTTCTGCT-3’ |
|  |  | cDNA_252 | 5’-CCCTACACGACGCTCTTCCGATCTNGTCTCATCAGGATCCCTGCTGCCTTGG-3’ | 5’-GACTGGAGTTCCTTGGCACCCGAGAATTCCACTCCCCTCCCGTCTTCTGCT-3’ |
|  |  | cDNA_253 | 5’-CCCTACACGACGCTCTTCCGATCTNACTAGATCAGGATCCCTGCTGCCTTGG-3’ | 5’-GACTGGAGTTCCTTGGCACCCGAGAATTCCACTCCCCTCCCGTCTTCTGCT-3’ |
|  |  | cDNA_254 | 5’-CCCTACACGACGCTCTTCCGATCTNGTGCTGACAGGATCCCTGCTGCCTTGG-3’ | 5’-GACTGGAGTTCCTTGGCACCCGAGAATTCCACTCCCCTCCCGTCTTCTGCT-3’ |
|  |  | cDNA_255 | 5’-CCCTACACGACGCTCTTCCGATCTNTACACGACAGGATCCCTGCTGCCTTGG-3’ | 5’-GACTGGAGTTCCTTGGCACCCGAGAATTCCACTCCCCTCCCGTCTTCTGCT-3’ |
|  |  | cDNA_256 | 5’-CCCTACACGACGCTCTTCCGATCTNGTCTCGCCAGGATCCCTGCTGCCTTGG-3’ | 5’-GACTGGAGTTCCTTGGCACCCGAGAATTCCACTCCCCTCCCGTCTTCTGCT-3’ |
|  |  | cDNA_257 | 5’-CCCTACACGACGCTCTTCCGATCTNTATATGCCAGGATCCCTGCTGCCTTGG-3’ | 5’-GACTGGAGTTCCTTGGCACCCGAGAATTCCACTCCCCTCCCGTCTTCTGCT-3’ |
|  |  | cDNA_258 | 5’-CCCTACACGACGCTCTTCCGATCTNGAGCTGCCAGGATCCCTGCTGCCTTGG-3’ | 5’-GACTGGAGTTCCTTGGCACCCGAGAATTCCACTCCCCTCCCGTCTTCTGCT-3’ |
|  |  | cDNA_259 | 5’-CCCTACACGACGCTCTTCCGATCTNGATCGATCAGGATCCCTGCTGCCTTGG-3’ | 5’-GACTGGAGTTCCTTGGCACCCGAGAATTCCACTCCCCTCCCGTCTTCTGCT-3’ |
|  |  | cDNA_260 | 5’-CCCTACACGACGCTCTTCCGATCTNTCACAGTCAGGATCCCTGCTGCCTTGG-3’ | 5’-GACTGGAGTTCCTTGGCACCCGAGAATTCCACTCCCCTCCCGTCTTCTGCT-3’ |
|  |  | cDNA_261 | 5’-CCCTACACGACGCTCTTCCGATCTNCTACGTACAGGATCCCTGCTGCCTTGG-3’ | 5’-GACTGGAGTTCCTTGGCACCCGAGAATTCCACTCCCCTCCCGTCTTCTGCT-3’ |
|  |  | cDNA_262 | 5’-CCCTACACGACGCTCTTCCGATCTNGCTGACTCAGGATCCCTGCTGCCTTGG-3’ | 5’-GACTGGAGTTCCTTGGCACCCGAGAATTCCACTCCCCTCCCGTCTTCTGCT-3’ |
|  |  | cDNA_263 | 5’-CCCTACACGACGCTCTTCCGATCTNAGATCGACAGGATCCCTGCTGCCTTGG-3’ | 5’-GACTGGAGTTCCTTGGCACCCGAGAATTCCACTCCCCTCCCGTCTTCTGCT-3’ |
|  |  | cDNA_264 | 5’-CCCTACACGACGCTCTTCCGATCTNGCGCAGTCAGGATCCCTGCTGCCTTGG-3’ | 5’-GACTGGAGTTCCTTGGCACCCGAGAATTCCACTCCCCTCCCGTCTTCTGCT-3’ |
|  |  | cDNA_265 | 5’-CCCTACACGACGCTCTTCCGATCTNGAGCACGCAGGATCCCTGCTGCCTTGG-3’ | 5’-GACTGGAGTTCCTTGGCACCCGAGAATTCCACTCCCCTCCCGTCTTCTGCT-3’ |
|  |  | cDNA_266 | 5’-CCCTACACGACGCTCTTCCGATCTNATATATCCAGGATCCCTGCTGCCTTGG-3’ | 5’-GACTGGAGTTCCTTGGCACCCGAGAATTCCACTCCCCTCCCGTCTTCTGCT-3’ |
|  |  | cDNA_267 | 5’-CCCTACACGACGCTCTTCCGATCTNGATCGTCCAGGATCCCTGCTGCCTTGG-3’ | 5’-GACTGGAGTTCCTTGGCACCCGAGAATTCCACTCCCCTCCCGTCTTCTGCT-3’ |
|  |  | cDNA_268 | 5’-CCCTACACGACGCTCTTCCGATCTNGTGTAGCCAGGATCCCTGCTGCCTTGG-3’ | 5’-GACTGGAGTTCCTTGGCACCCGAGAATTCCACTCCCCTCCCGTCTTCTGCT-3’ |
|  |  | cDNA_269 | 5’-CCCTACACGACGCTCTTCCGATCTNTACGTGTCAGGATCCCTGCTGCCTTGG-3’ | 5’-GACTGGAGTTCCTTGGCACCCGAGAATTCCACTCCCCTCCCGTCTTCTGCT-3’ |
|  |  | cDNA_270 | 5’-CCCTACACGACGCTCTTCCGATCTNTGATGTCCAGGATCCCTGCTGCCTTGG-3’ | 5’-GACTGGAGTTCCTTGGCACCCGAGAATTCCACTCCCCTCCCGTCTTCTGCT-3’ |
|  |  |  |  |  |
|  |  |  |  |  |

**Supplementary Table 2****. Quality and data filtering of 35 paired cervical samples.**

| **Samples** | **Raw reads** | **Raw bases** | **Length** | **Err(%)** | **GC%** | **Q20(%)** | **Q30(%)** | **Rm Adapter** | **Rm N** | **Low quality** | **Clean reads** | **Clean bases** | **Clean Q30** | **Clean rate(%)** |
| --- | --- | --- | --- | --- | --- | --- | --- | --- | --- | --- | --- | --- | --- | --- |
| 1-10630-1_r1 | 114407 | 34322100 | 300 | 0.48 | 60.43 | 94.93 | 90.32 | 0 | 0 | 9536 | 96061 | 28818139 | 94.70 | 83.96 |
| 1-10630-1_r2 | 114407 | 34322100 | 300 | 1.36 | 57.24 | 86.19 | 76.76 | 1624 | 0 | 11463 | 96061 | 28350534 | 82.96 | 82.60 |
| 2-10630-2_r1 | 104218 | 31265400 | 300 | 0.65 | 59.14 | 93.34 | 88.59 | 0 | 0 | 12265 | 84508 | 25352133 | 94.17 | 81.09 |
| 2-10630-2_r2 | 104218 | 31265400 | 300 | 1.53 | 56.46 | 84.79 | 75.55 | 3510 | 0 | 10101 | 84508 | 24447360 | 83.75 | 78.19 |
| 3-11180-1_r1 | 93965 | 28189500 | 300 | 0.35 | 61.96 | 96.22 | 91.95 | 0 | 0 | 4729 | 81830 | 24548949 | 95.34 | 87.09 |
| 3-11180-1_r2 | 93965 | 28189500 | 300 | 1.22 | 58.03 | 87.31 | 77.94 | 990 | 0 | 9366 | 81830 | 24317700 | 82.93 | 86.27 |
| 4-11180-2_r1 | 116053 | 34815900 | 300 | 0.62 | 61.14 | 93.44 | 88.89 | 0 | 0 | 13024 | 94383 | 28314842 | 95.10 | 81.33 |
| 4-11180-2_r2 | 116053 | 34815900 | 300 | 1.53 | 57.46 | 84.56 | 75.41 | 8412 | 0 | 10704 | 94383 | 27831802 | 83.15 | 79.94 |
| 5-12035-1_r1 | 102235 | 30670500 | 300 | 0.25 | 63.44 | 97.21 | 93.29 | 0 | 0 | 2860 | 91177 | 27353081 | 95.87 | 89.18 |
| 5-12035-1_r2 | 102235 | 30670500 | 300 | 1.13 | 58.77 | 88.09 | 78.81 | 901 | 0 | 9716 | 91177 | 27311595 | 82.70 | 89.05 |
| 6-12035-2_r1 | 98991 | 29697300 | 300 | 0.37 | 61.93 | 96.02 | 91.59 | 0 | 0 | 5200 | 85715 | 25714411 | 95.15 | 86.59 |
| 6-12035-2_r2 | 98991 | 29697300 | 300 | 1.23 | 57.81 | 87.20 | 77.75 | 1573 | 0 | 10116 | 85715 | 25529685 | 82.76 | 85.97 |
| 7-12310-1_r1 | 104580 | 31374000 | 300 | 0.40 | 61.46 | 95.70 | 91.33 | 0 | 0 | 6354 | 89705 | 26911480 | 95.26 | 85.78 |
| 7-12310-1_r2 | 104580 | 31374000 | 300 | 1.29 | 57.53 | 86.75 | 77.39 | 2248 | 0 | 10813 | 89705 | 26601468 | 82.97 | 84.79 |
| 8-12310-2_r1 | 120319 | 36095700 | 300 | 0.72 | 60.29 | 92.37 | 87.67 | 2 | 0 | 16763 | 94703 | 28410833 | 95.16 | 78.71 |
| 8-12310-2_r2 | 120319 | 36095700 | 300 | 1.64 | 57.16 | 83.49 | 74.35 | 11656 | 0 | 10907 | 94703 | 27982577 | 83.02 | 77.52 |
| 9-13048-1_r1 | 94925 | 28477500 | 300 | 0.47 | 61.55 | 94.97 | 90.55 | 0 | 0 | 7387 | 79932 | 23979531 | 95.36 | 84.21 |
| 9-13048-1_r2 | 94925 | 28477500 | 300 | 1.36 | 57.66 | 86.06 | 76.69 | 4118 | 0 | 9311 | 79932 | 23762164 | 82.85 | 83.44 |
| 10-13048-2_r1 | 115925 | 34777500 | 300 | 0.42 | 62.43 | 95.47 | 90.88 | 0 | 0 | 7338 | 99484 | 29845159 | 94.99 | 85.82 |
| 10-13048-2_r2 | 115925 | 34777500 | 300 | 1.29 | 58.15 | 86.65 | 77.32 | 3529 | 0 | 11345 | 99484 | 29605741 | 82.83 | 85.13 |
| 11-13202-1_r1 | 106808 | 32042400 | 300 | 0.31 | 61.77 | 96.58 | 92.10 | 0 | 0 | 4041 | 93724 | 28117172 | 95.13 | 87.75 |
| 11-13202-1_r2 | 106808 | 32042400 | 300 | 1.17 | 57.91 | 87.70 | 78.11 | 906 | 0 | 11179 | 93724 | 28007396 | 82.54 | 87.41 |
| 12-13202-2_r1 | 120716 | 36214800 | 300 | 0.72 | 59.96 | 92.34 | 87.54 | 2 | 0 | 17029 | 94538 | 28361345 | 94.99 | 78.31 |
| 12-13202-2_r2 | 120716 | 36214800 | 300 | 1.64 | 56.88 | 83.53 | 74.28 | 10632 | 0 | 11358 | 94538 | 27912304 | 82.93 | 77.07 |
| 13-13305-1_r1 | 99714 | 29914200 | 300 | 0.50 | 60.69 | 94.68 | 90.20 | 0 | 0 | 8657 | 83325 | 24997396 | 95.05 | 83.56 |
| 13-13305-1_r2 | 99714 | 29914200 | 300 | 1.39 | 57.49 | 85.81 | 76.52 | 3734 | 0 | 9622 | 83325 | 24669094 | 82.92 | 82.47 |
| 14-13305-2_r1 | 123542 | 37062600 | 300 | 1.04 | 58.92 | 88.96 | 83.71 | 11 | 0 | 26891 | 88332 | 26499441 | 94.87 | 71.50 |
| 14-13305-2_r2 | 123542 | 37062600 | 300 | 1.97 | 56.34 | 80.33 | 71.31 | 20541 | 0 | 10583 | 88332 | 26012999 | 83.12 | 70.19 |
| 15-13389-1_r1 | 90623 | 27186900 | 300 | 0.29 | 61.10 | 96.80 | 92.42 | 1 | 0 | 3028 | 78859 | 23657700 | 95.42 | 87.02 |
| 15-13389-1_r2 | 90623 | 27186900 | 300 | 1.18 | 57.82 | 87.51 | 77.68 | 666 | 0 | 10529 | 78859 | 23599408 | 82.26 | 86.80 |
| 16-13389-2_r1 | 122115 | 36634500 | 300 | 0.81 | 59.64 | 91.42 | 86.48 | 1 | 0 | 19676 | 93471 | 28041269 | 95.02 | 76.54 |
| 16-13389-2_r2 | 122115 | 36634500 | 300 | 1.73 | 56.92 | 82.62 | 73.44 | 13824 | 0 | 11271 | 93471 | 27638901 | 82.89 | 75.45 |
| 17-13842-1_r1 | 64192 | 19257600 | 300 | 0.25 | 64.23 | 97.10 | 92.71 | 0 | 0 | 1586 | 56646 | 16993800 | 95.31 | 88.24 |
| 17-13842-1_r2 | 64192 | 19257600 | 300 | 1.16 | 58.86 | 87.81 | 78.23 | 161 | 0 | 7146 | 56646 | 16978906 | 82.34 | 88.17 |
| 18-13842-2_r1 | 89162 | 26748600 | 300 | 0.29 | 62.63 | 96.76 | 92.45 | 0 | 0 | 3057 | 78701 | 23610288 | 95.32 | 88.27 |
| 18-13842-2_r2 | 89162 | 26748600 | 300 | 1.16 | 58.35 | 87.82 | 78.37 | 805 | 0 | 9037 | 78701 | 23532450 | 82.63 | 87.98 |
| 19-13964-1_r1 | 98410 | 29523000 | 300 | 0.32 | 63.01 | 96.49 | 92.30 | 1 | 0 | 4232 | 86099 | 25829687 | 95.60 | 87.49 |
| 19-13964-1_r2 | 98410 | 29523000 | 300 | 1.20 | 58.37 | 87.44 | 78.09 | 1633 | 0 | 9871 | 86099 | 25720003 | 82.81 | 87.12 |
| 20-13964-2_r1 | 105470 | 31641000 | 300 | 0.40 | 61.79 | 95.65 | 91.07 | 0 | 0 | 6275 | 90271 | 27081253 | 95.10 | 85.59 |
| 20-13964-2_r2 | 105470 | 31641000 | 300 | 1.29 | 57.80 | 86.65 | 77.07 | 2638 | 0 | 11013 | 90271 | 26886918 | 82.57 | 84.97 |
| 21-13991-1_r1 | 94311 | 28293300 | 300 | 0.44 | 61.78 | 95.24 | 90.75 | 0 | 0 | 6716 | 79997 | 23999062 | 95.10 | 84.82 |
| 21-13991-1_r2 | 94311 | 28293300 | 300 | 1.33 | 57.80 | 86.30 | 76.93 | 2994 | 0 | 9402 | 79997 | 23744267 | 82.86 | 83.92 |
| 22-13991-2_r1 | 118938 | 35681400 | 300 | 1.00 | 59.68 | 89.61 | 84.57 | 2 | 0 | 24228 | 86871 | 26061117 | 94.30 | 73.04 |
| 22-13991-2_r2 | 118938 | 35681400 | 300 | 1.96 | 56.66 | 80.81 | 71.88 | 14826 | 0 | 10217 | 86871 | 25135509 | 83.43 | 70.44 |
| 23-14140-1_r1 | 91426 | 27427800 | 300 | 0.33 | 62.68 | 96.29 | 91.69 | 0 | 0 | 3822 | 79991 | 23997287 | 94.90 | 87.49 |
| 23-14140-1_r2 | 91426 | 27427800 | 300 | 1.19 | 57.76 | 87.55 | 78.03 | 1162 | 0 | 9414 | 79991 | 23868662 | 82.73 | 87.02 |
| 24-14140-2_r1 | 109827 | 32948100 | 300 | 0.29 | 62.50 | 96.74 | 92.43 | 0 | 0 | 3767 | 96779 | 29033694 | 95.30 | 88.12 |
| 24-14140-2_r2 | 109827 | 32948100 | 300 | 1.17 | 58.29 | 87.72 | 78.24 | 1078 | 0 | 11176 | 96779 | 28905240 | 82.57 | 87.73 |
| 25-14224-1_r1 | 109323 | 32796900 | 300 | 0.50 | 61.13 | 94.68 | 90.24 | 0 | 0 | 9367 | 91628 | 27488370 | 95.19 | 83.81 |
| 25-14224-1_r2 | 109323 | 32796900 | 300 | 1.38 | 57.57 | 85.86 | 76.60 | 4894 | 0 | 10302 | 91628 | 27186621 | 83.02 | 82.89 |
| 26-14224-2_r1 | 104680 | 31404000 | 300 | 0.81 | 59.78 | 91.51 | 86.64 | 0 | 0 | 16931 | 80210 | 24062925 | 94.75 | 76.62 |
| 26-14224-2_r2 | 104680 | 31404000 | 300 | 1.73 | 56.83 | 82.72 | 73.59 | 10130 | 0 | 9465 | 80210 | 23563157 | 83.04 | 75.03 |
| 27-14265-1_r1 | 108979 | 32693700 | 300 | 0.54 | 60.89 | 94.30 | 89.66 | 0 | 0 | 10205 | 90531 | 27159207 | 94.77 | 83.07 |
| 27-14265-1_r2 | 108979 | 32693700 | 300 | 1.44 | 57.33 | 85.47 | 76.21 | 4213 | 0 | 10556 | 90531 | 26569846 | 83.35 | 81.27 |
| 28-14265-2_r1 | 91296 | 27388800 | 300 | 0.38 | 61.47 | 95.89 | 91.38 | 0 | 0 | 4941 | 78471 | 23541264 | 95.12 | 85.95 |
| 28-14265-2_r2 | 91296 | 27388800 | 300 | 1.25 | 57.46 | 86.98 | 77.41 | 1628 | 0 | 9900 | 78471 | 23328215 | 82.83 | 85.17 |
| 29-14793-1_r1 | 117357 | 35207100 | 300 | 0.35 | 61.94 | 96.13 | 91.87 | 0 | 0 | 6024 | 101952 | 30585582 | 95.44 | 86.87 |
| 29-14793-1_r2 | 117357 | 35207100 | 300 | 1.24 | 58.30 | 87.12 | 77.82 | 2411 | 0 | 11522 | 101952 | 30407730 | 82.77 | 86.37 |
| 30-14793-2_r1 | 61307 | 18392100 | 300 | 0.29 | 63.23 | 96.76 | 92.72 | 0 | 0 | 2302 | 54144 | 16243188 | 95.62 | 88.32 |
| 30-14793-2_r2 | 61307 | 18392100 | 300 | 1.17 | 58.53 | 87.72 | 78.41 | 953 | 0 | 5755 | 54144 | 16192722 | 82.63 | 88.04 |
| 31-15357-1_r1 | 114487 | 34346100 | 300 | 0.29 | 63.10 | 96.81 | 92.64 | 0 | 0 | 3945 | 101447 | 30434068 | 95.44 | 88.61 |
| 31-15357-1_r2 | 114487 | 34346100 | 300 | 1.16 | 58.63 | 87.84 | 78.53 | 1188 | 0 | 10909 | 101447 | 30326931 | 82.69 | 88.30 |
| 32-15357-2_r1 | 101341 | 30402300 | 300 | 0.26 | 62.90 | 97.13 | 93.17 | 0 | 0 | 2949 | 90412 | 27123594 | 95.71 | 89.22 |
| 32-15357-2_r2 | 101341 | 30402300 | 300 | 1.13 | 58.67 | 88.14 | 78.92 | 820 | 0 | 9505 | 90412 | 27052627 | 82.82 | 88.98 |
| 33-13351-1_r1 | 122683 | 36804900 | 300 | 1.17 | 57.93 | 87.78 | 82.62 | 4 | 0 | 31021 | 84345 | 25303352 | 94.43 | 68.75 |
| 33-13351-1_r2 | 122683 | 36804900 | 300 | 2.11 | 55.72 | 79.34 | 70.62 | 19853 | 0 | 9563 | 84345 | 24496572 | 83.54 | 66.56 |
| 34-13351-2_r1 | 126345 | 37903500 | 300 | 0.88 | 59.65 | 90.76 | 85.84 | 4 | 0 | 22706 | 95159 | 28547519 | 94.77 | 75.32 |
| 34-13351-2_r2 | 126345 | 37903500 | 300 | 1.80 | 56.65 | 82.14 | 73.15 | 14095 | 0 | 11049 | 95159 | 27855162 | 83.44 | 73.49 |
| 35-03157-1_r1 | 114776 | 34432800 | 300 | 0.62 | 61.12 | 93.47 | 88.91 | 0 | 0 | 13220 | 93418 | 28025307 | 95.02 | 81.39 |
| 35-03157-1_r2 | 114776 | 34432800 | 300 | 1.51 | 57.51 | 84.75 | 75.68 | 7638 | 0 | 10044 | 93418 | 27653018 | 83.03 | 80.31 |
| 36-03157-2_r1 | 146719 | 44015700 | 300 | 0.81 | 60.65 | 91.46 | 86.68 | 0 | 0 | 23916 | 112240 | 33671861 | 94.91 | 76.50 |
| 36-03157-2_r2 | 146719 | 44015700 | 300 | 1.75 | 57.08 | 82.57 | 73.50 | 14532 | 0 | 13417 | 112240 | 32967879 | 83.03 | 74.90 |
| 37-JL-1_r1 | 135878 | 40763400 | 300 | 0.70 | 60.32 | 92.61 | 87.90 | 1 | 0 | 18014 | 108352 | 32505440 | 94.71 | 79.74 |
| 37-JL-1_r2 | 135878 | 40763400 | 300 | 1.60 | 56.99 | 83.97 | 74.92 | 10917 | 0 | 11924 | 108352 | 31771686 | 83.35 | 77.94 |
| 38-JL-2_r1 | 132129 | 39638700 | 300 | 0.39 | 62.27 | 95.74 | 91.52 | 0 | 0 | 8106 | 113343 | 34002806 | 95.52 | 85.78 |
| 38-JL-2_r2 | 132129 | 39638700 | 300 | 1.30 | 58.21 | 86.60 | 77.28 | 3644 | 0 | 13081 | 113343 | 33679361 | 82.81 | 84.97 |
| 39-LQ-1_r1 | 129461 | 38838300 | 300 | 0.46 | 61.27 | 95.05 | 90.52 | 0 | 0 | 9787 | 109138 | 32741289 | 95.11 | 84.30 |
| 39-LQ-1_r2 | 129461 | 38838300 | 300 | 1.35 | 57.80 | 86.08 | 76.68 | 4791 | 0 | 13012 | 109138 | 32403725 | 82.75 | 83.43 |
| 40-LQ-2_r1 | 132759 | 39827700 | 300 | 0.34 | 62.25 | 96.26 | 91.91 | 0 | 0 | 6372 | 115121 | 34536282 | 95.48 | 86.71 |
| 40-LQ-2_r2 | 132759 | 39827700 | 300 | 1.22 | 57.96 | 87.27 | 77.77 | 2838 | 0 | 13694 | 115121 | 34402607 | 82.71 | 86.38 |
| 41-BYQ-1_r1 | 120254 | 36076200 | 300 | 0.42 | 62.57 | 95.42 | 91.02 | 0 | 0 | 8265 | 102103 | 30630841 | 95.37 | 84.91 |
| 41-BYQ-1_r2 | 120254 | 36076200 | 300 | 1.33 | 58.10 | 86.35 | 76.95 | 3679 | 0 | 12407 | 102103 | 30340025 | 82.82 | 84.10 |
| 42-BYQ-2_r1 | 130304 | 39091200 | 300 | 0.48 | 62.27 | 94.85 | 90.40 | 0 | 0 | 10505 | 109773 | 32931858 | 95.21 | 84.24 |
| 42-BYQ-2_r2 | 130304 | 39091200 | 300 | 1.37 | 57.91 | 85.95 | 76.65 | 5974 | 0 | 12165 | 109773 | 32676030 | 82.70 | 83.59 |
| 43-DYZ-1_r1 | 114655 | 34396500 | 300 | 0.22 | 64.37 | 97.46 | 93.49 | 0 | 0 | 2404 | 102828 | 30848400 | 95.78 | 89.68 |
| 43-DYZ-1_r2 | 114655 | 34396500 | 300 | 1.10 | 58.98 | 88.33 | 78.96 | 298 | 0 | 11256 | 102828 | 30835529 | 82.64 | 89.65 |
| 44-DYZ-2_r1 | 135847 | 40754100 | 300 | 0.38 | 62.75 | 95.82 | 91.55 | 1 | 0 | 7932 | 116629 | 34988672 | 95.59 | 85.85 |
| 44-DYZ-2_r2 | 135847 | 40754100 | 300 | 1.28 | 58.27 | 86.74 | 77.35 | 4313 | 0 | 13619 | 116629 | 34855794 | 82.69 | 85.53 |
| 45-HJR-1_r1 | 148273 | 44481900 | 300 | 0.60 | 60.78 | 93.58 | 88.98 | 0 | 0 | 16477 | 120471 | 36141099 | 95.14 | 81.25 |
| 45-HJR-1_r2 | 148273 | 44481900 | 300 | 1.50 | 57.47 | 84.72 | 75.47 | 10078 | 0 | 13997 | 120471 | 35684382 | 82.88 | 80.22 |
| 46-HJR-2_r1 | 129063 | 38718900 | 300 | 0.34 | 62.63 | 96.29 | 92.11 | 0 | 0 | 6148 | 112716 | 33814751 | 95.57 | 87.33 |
| 46-HJR-2_r2 | 129063 | 38718900 | 300 | 1.21 | 58.40 | 87.38 | 78.03 | 3009 | 0 | 12384 | 112716 | 33711842 | 82.75 | 87.07 |
| 47-HXQ-1_r1 | 135171 | 40551300 | 300 | 1.75 | 56.44 | 81.73 | 75.82 | 0 | 0 | 52548 | 76033 | 22809697 | 94.04 | 56.25 |
| 47-HXQ-1_r2 | 135171 | 40551300 | 300 | 2.75 | 54.84 | 73.53 | 65.04 | 40490 | 0 | 9075 | 76033 | 21795870 | 84.06 | 53.75 |
| 48-HXQ-2_r1 | 151675 | 45502500 | 300 | 1.57 | 56.68 | 83.70 | 78.01 | 2 | 0 | 52252 | 91378 | 27413060 | 93.95 | 60.25 |
| 48-HXQ-2_r2 | 151675 | 45502500 | 300 | 2.55 | 55.06 | 75.31 | 66.71 | 37694 | 0 | 11139 | 91378 | 26145411 | 84.02 | 57.46 |
| 49-LJF-1_r1 | 138955 | 41686500 | 300 | 1.62 | 56.21 | 83.17 | 77.44 | 1 | 0 | 49772 | 82058 | 24617175 | 93.88 | 59.05 |
| 49-LJF-1_r2 | 138955 | 41686500 | 300 | 2.60 | 54.78 | 74.83 | 66.27 | 36290 | 0 | 9984 | 82058 | 23384936 | 84.15 | 56.10 |
| 50-LJF-2_r1 | 130619 | 39185700 | 300 | 1.14 | 58.60 | 88.09 | 82.73 | 1 | 0 | 31225 | 90905 | 27271351 | 94.38 | 69.60 |
| 50-LJF-2_r2 | 130619 | 39185700 | 300 | 2.08 | 56.12 | 79.56 | 70.59 | 21880 | 0 | 11230 | 90905 | 26434770 | 83.48 | 67.46 |
| 51-LXY-1_r1 | 119271 | 35781300 | 300 | 0.46 | 62.37 | 95.00 | 90.66 | 0 | 0 | 9238 | 100576 | 30172781 | 95.48 | 84.33 |
| 51-LXY-1_r2 | 119271 | 35781300 | 300 | 1.36 | 58.10 | 85.98 | 76.70 | 5557 | 0 | 11487 | 100576 | 29941744 | 82.80 | 83.68 |
| 52-LXY-2_r1 | 128730 | 38619000 | 300 | 1.00 | 58.86 | 89.57 | 84.59 | 2 | 0 | 26926 | 93741 | 28122113 | 94.58 | 72.82 |
| 52-LXY-2_r2 | 128730 | 38619000 | 300 | 1.93 | 56.28 | 81.00 | 72.13 | 17429 | 0 | 10315 | 93741 | 27309996 | 83.56 | 70.72 |
| 53-LYP-1_r1 | 141712 | 42513600 | 300 | 0.65 | 60.64 | 93.06 | 88.44 | 0 | 0 | 17554 | 113443 | 34032780 | 95.20 | 80.05 |
| 53-LYP-1_r2 | 141712 | 42513600 | 300 | 1.56 | 57.34 | 84.24 | 75.08 | 11091 | 0 | 13332 | 113443 | 33533001 | 83.10 | 78.88 |
| 54-LYP-2_r1 | 134230 | 40269000 | 300 | 1.18 | 58.47 | 87.65 | 82.46 | 1 | 0 | 33819 | 91996 | 27598672 | 94.51 | 68.54 |
| 54-LYP-2_r2 | 134230 | 40269000 | 300 | 2.14 | 55.99 | 79.06 | 70.25 | 23371 | 0 | 10994 | 91996 | 26699353 | 83.53 | 66.30 |
| 55-MWZ-1_r1 | 139496 | 41848800 | 300 | 0.41 | 62.31 | 95.53 | 90.97 | 0 | 0 | 8547 | 120245 | 36073421 | 94.89 | 86.20 |
| 55-MWZ-1_r2 | 139496 | 41848800 | 300 | 1.28 | 58.18 | 86.82 | 77.56 | 3550 | 0 | 13536 | 120245 | 35732924 | 82.97 | 85.39 |
| 56-MWZ-2_r1 | 137375 | 41212500 | 300 | 1.68 | 56.45 | 82.62 | 76.80 | 3 | 0 | 51104 | 79847 | 23953690 | 93.52 | 58.12 |
| 56-MWZ-2_r2 | 137375 | 41212500 | 300 | 2.67 | 54.89 | 74.37 | 65.96 | 35906 | 0 | 9356 | 79847 | 22694780 | 84.37 | 55.07 |
| 57-ZYX-1_r1 | 131925 | 39577500 | 300 | 0.30 | 62.67 | 96.71 | 92.57 | 0 | 0 | 4909 | 116683 | 35004884 | 95.52 | 88.45 |
| 57-ZYX-1_r2 | 131925 | 39577500 | 300 | 1.16 | 58.51 | 87.81 | 78.53 | 1898 | 0 | 12510 | 116683 | 34896951 | 82.77 | 88.17 |
| 58-ZYX_r1 | 115981 | 34794300 | 300 | 0.30 | 63.82 | 96.62 | 92.45 | 0 | 0 | 4410 | 102121 | 30636294 | 95.54 | 88.05 |
| 58-ZYX_r2 | 115981 | 34794300 | 300 | 1.19 | 58.67 | 87.57 | 78.23 | 1945 | 0 | 11324 | 102121 | 30581766 | 82.55 | 87.89 |
| 59-824687-1_r1 | 143855 | 43156500 | 300 | 1.15 | 58.75 | 87.84 | 82.61 | 2 | 0 | 34767 | 100032 | 30009440 | 94.89 | 69.54 |
| 59-824687-1_r2 | 143855 | 43156500 | 300 | 2.08 | 56.14 | 79.34 | 70.46 | 27431 | 0 | 11544 | 100032 | 29371812 | 83.34 | 68.06 |
| 60-824687-2_r1 | 161890 | 48567000 | 300 | 1.89 | 54.94 | 80.28 | 74.13 | 2 | 0 | 67365 | 86931 | 26079038 | 93.60 | 53.70 |
| 60-824687-2_r2 | 161890 | 48567000 | 300 | 2.88 | 54.01 | 72.10 | 63.66 | 53993 | 0 | 10652 | 86931 | 24555939 | 84.49 | 50.56 |
| 61-825333-1_r1 | 152699 | 45809700 | 300 | 1.85 | 55.36 | 80.64 | 74.39 | 1 | 0 | 62677 | 81949 | 24584480 | 93.96 | 53.67 |
| 61-825333-1_r2 | 152699 | 45809700 | 300 | 2.85 | 54.22 | 72.31 | 63.67 | 50484 | 0 | 10989 | 81949 | 23384723 | 84.07 | 51.05 |
| 62-825333-2_r1 | 161780 | 48534000 | 300 | 1.75 | 56.06 | 81.67 | 75.65 | 2 | 0 | 62119 | 91248 | 27374189 | 93.99 | 56.40 |
| 62-825333-2_r2 | 161780 | 48534000 | 300 | 2.74 | 54.61 | 73.44 | 64.86 | 49838 | 0 | 11267 | 91248 | 26162669 | 83.95 | 53.91 |
| 63-828968-1_r1 | 127016 | 38104800 | 300 | 0.46 | 62.46 | 94.96 | 90.66 | 0 | 0 | 9844 | 107715 | 32314408 | 95.52 | 84.80 |
| 63-828968-1_r2 | 127016 | 38104800 | 300 | 1.34 | 58.23 | 86.14 | 76.98 | 6616 | 0 | 11422 | 107715 | 32149407 | 82.90 | 84.37 |
| 64-828968-2_r1 | 149118 | 44735400 | 300 | 0.94 | 60.46 | 90.05 | 85.16 | 0 | 0 | 28473 | 110288 | 33086286 | 95.11 | 73.96 |
| 64-828968-2_r2 | 149118 | 44735400 | 300 | 1.89 | 56.92 | 81.25 | 72.31 | 21804 | 0 | 12668 | 110288 | 32450328 | 83.19 | 72.54 |
| 65-829197-1_r1 | 121181 | 36354300 | 300 | 0.30 | 63.98 | 96.67 | 92.56 | 0 | 0 | 4686 | 106725 | 32017492 | 95.66 | 88.07 |
| 65-829197-1_r2 | 121181 | 36354300 | 300 | 1.18 | 58.74 | 87.64 | 78.32 | 1878 | 0 | 11951 | 106725 | 31964257 | 82.63 | 87.92 |
| 66-829197-2_r1 | 146593 | 43977900 | 300 | 1.10 | 58.06 | 88.41 | 83.30 | 0 | 0 | 34529 | 102914 | 30874077 | 94.77 | 70.20 |
| 66-829197-2_r2 | 146593 | 43977900 | 300 | 2.03 | 55.83 | 79.93 | 71.05 | 24211 | 0 | 11646 | 102914 | 30142875 | 83.28 | 68.54 |
| 67-837251-1_r1 | 126884 | 38065200 | 300 | 0.62 | 61.25 | 93.39 | 88.81 | 2 | 0 | 14634 | 102797 | 30839035 | 95.26 | 81.02 |
| 67-837251-1_r2 | 126884 | 38065200 | 300 | 1.51 | 57.34 | 84.62 | 75.41 | 9677 | 0 | 11606 | 102797 | 30568374 | 82.87 | 80.31 |
| 68-837251-2_r1 | 118760 | 35628000 | 300 | 1.04 | 58.92 | 89.22 | 84.18 | 1 | 0 | 25586 | 85176 | 25552668 | 94.51 | 71.72 |
| 68-837251-2_r2 | 118760 | 35628000 | 300 | 1.99 | 56.24 | 80.47 | 71.52 | 16395 | 0 | 10546 | 85176 | 24697007 | 83.55 | 69.32 |
| 69-13502-1_r1 | 132804 | 39841200 | 300 | 1.72 | 55.86 | 82.05 | 76.14 | 1 | 0 | 50221 | 76011 | 22803156 | 93.85 | 57.24 |
| 69-13502-1_r2 | 132804 | 39841200 | 300 | 2.71 | 54.52 | 73.75 | 65.26 | 39431 | 0 | 9159 | 76011 | 21654761 | 84.27 | 54.35 |
| 70-13502-2_r1 | 174664 | 52399200 | 300 | 1.98 | 54.54 | 79.34 | 73.29 | 2 | 0 | 77329 | 89940 | 26981662 | 93.70 | 51.49 |
| 70-13502-2_r2 | 174664 | 52399200 | 300 | 2.97 | 53.62 | 71.35 | 63.12 | 61568 | 0 | 10540 | 89940 | 25411988 | 84.55 | 48.50 |

**Supplementary Table 4. Numbers of reads classified into eight cases in high-throughput sequencing database.**

| **Samples** | **5 A>G** | **14 A>G** | **44 A>G** | **5 A>G+14 A>G** | **5 A>G+44 A>G** | **14 A>G+44 A>G** | **5 A>G+14 A>G+44 A>G** | **Wild type** |
| --- | --- | --- | --- | --- | --- | --- | --- | --- |
| 1-10630-1 | 3685 | 238 | 163 | 3802 | 753 | 31 | 1761 | 59880 |
| 2-10630-2 | 2976 | 136 | 113 | 2778 | 660 | 14 | 1253 | 45322 |
| 3-11180-1 | 3328 | 235 | 115 | 2998 | 578 | 8 | 1388 | 57154 |
| 4-11180-2 | 2764 | 130 | 209 | 3405 | 640 | 16 | 1683 | 65912 |
| 5-12035-1 | 3827 | 245 | 245 | 4595 | 808 | 35 | 2863 | 68664 |
| 6-12035-2 | 3021 | 208 | 143 | 3270 | 579 | 12 | 1587 | 35740 |
| 7-12310-1 | 6148 | 246 | 276 | 9855 | 2586 | 70 | 6396 | 45197 |
| 8-12310-2 | 3198 | 282 | 230 | 3208 | 758 | 19 | 1375 | 67876 |
| 9-13048-1 | 3279 | 222 | 109 | 3385 | 680 | 35 | 1599 | 53707 |
| 10-13048-2 | 3092 | 234 | 216 | 3784 | 934 | 11 | 1516 | 50570 |
| 11-13202-1 | 3504 | 235 | 136 | 3188 | 696 | 14 | 1783 | 42085 |
| 12-13202-2 | 3423 | 228 | 197 | 3921 | 716 | 31 | 1675 | 61850 |
| 13-13305-1 | 2923 | 177 | 240 | 2957 | 629 | 10 | 1341 | 56168 |
| 14-13305-2 | 2826 | 167 | 136 | 2594 | 281 | 4 | 778 | 64574 |
| 15-13389-1 | 2236 | 237 | 137 | 3446 | 624 | 8 | 1423 | 50869 |
| 16-13389-2 | 3173 | 139 | 250 | 3215 | 582 | 51 | 1218 | 67672 |
| 17-13842-1 | 2554 | 108 | 106 | 2840 | 656 | 15 | 1811 | 41998 |
| 18-13842-2 | 2778 | 183 | 98 | 3180 | 553 | 19 | 1586 | 57638 |
| 19-13964-1 | 8027 | 289 | 247 | 6251 | 1600 | 49 | 2324 | 54261 |
| 20-13964-2 | 3284 | 294 | 211 | 4011 | 681 | 34 | 1988 | 59382 |
| 21-13991-1 | 5301 | 252 | 229 | 6266 | 1358 | 31 | 2761 | 46297 |
| 22-13991-2 | 2955 | 218 | 296 | 3030 | 657 | 32 | 1477 | 56409 |
| 23-14140-1 | 4789 | 199 | 141 | 5875 | 1189 | 30 | 2591 | 48111 |
| 24-14140-2 | 4699 | 192 | 163 | 5227 | 1141 | 28 | 1951 | 66924 |
| 25-14224-1 | 3797 | 216 | 196 | 3672 | 804 | 41 | 1537 | 61825 |
| 26-14224-2 | 2899 | 200 | 259 | 2170 | 625 | 20 | 530 | 54549 |
| 27-14265-1 | 3588 | 236 | 180 | 3618 | 860 | 53 | 1694 | 57030 |
| 28-14265-2 | 3542 | 223 | 152 | 4712 | 968 | 30 | 2482 | 44785 |
| 29-14793-1 | 2597 | 186 | 130 | 2676 | 458 | 21 | 1206 | 53116 |
| 30-14793-2 | 1760 | 98 | 86 | 1520 | 392 | 17 | 680 | 42637 |
| 31-15357-1 | 3435 | 198 | 166 | 3222 | 599 | 22 | 1343 | 29047 |
| 32-15357-2 | 2158 | 159 | 99 | 2016 | 360 | 15 | 812 | 45135 |
| 33-13351-1 | 3558 | 246 | 211 | 3578 | 845 | 28 | 1709 | 54346 |
| 34-13351-2 | 3293 | 271 | 163 | 3483 | 764 | 57 | 1817 | 62140 |
| 35-03157-1 | 3504 | 250 | 227 | 3455 | 637 | 41 | 1797 | 66989 |
| 36-03157-2 | 4174 | 326 | 249 | 3819 | 738 | 52 | 1854 | 76755 |
| 37-JL-1 | 3198 | 189 | 317 | 2984 | 712 | 30 | 1383 | 72822 |
| 38-JL-2 | 3004 | 275 | 152 | 2902 | 631 | 26 | 1055 | 87129 |
| 39-LQ-1 | 2908 | 257 | 171 | 3137 | 735 | 28 | 1169 | 79381 |
| 40-LQ-2 | 2785 | 205 | 532 | 3560 | 548 | 6 | 1756 | 84499 |
| 41-BYQ-1 | 3593 | 239 | 230 | 3931 | 756 | 39 | 1929 | 72777 |
| 42-BYQ-2 | 4892 | 369 | 225 | 4853 | 1060 | 27 | 2539 | 75535 |
| 43-DYZ-1 | 2388 | 128 | 155 | 2894 | 595 | 34 | 1751 | 63167 |
| 44-DYZ-2 | 3558 | 214 | 216 | 3557 | 887 | 18 | 2223 | 89544 |
| 45-HJR-1 | 4953 | 366 | 240 | 4751 | 925 | 77 | 1862 | 84696 |
| 46-HJR-2 | 4111 | 286 | 139 | 3954 | 656 | 6 | 843 | 86115 |
| 47-HXQ-1 | 2450 | 204 | 195 | 2530 | 425 | 28 | 1099 | 48113 |
| 48-HXQ-2 | 3266 | 256 | 224 | 2778 | 565 | 39 | 1089 | 56513 |
| 49-LJF-1 | 2764 | 191 | 186 | 2372 | 586 | 35 | 926 | 49430 |
| 50-LJF-2 | 2520 | 147 | 248 | 2609 | 628 | 36 | 1125 | 39089 |
| 51-LXY-1 | 3169 | 197 | 170 | 3189 | 595 | 24 | 1486 | 45849 |
| 52-LXY-2 | 2939 | 255 | 146 | 2543 | 676 | 23 | 1325 | 62867 |
| 53-LYP-1 | 4652 | 254 | 271 | 4677 | 1068 | 32 | 2785 | 55060 |
| 54-LYP-2 | 2962 | 222 | 173 | 2752 | 485 | 29 | 1216 | 61045 |
| 55-MWZ-1 | 4543 | 264 | 226 | 4585 | 850 | 35 | 2242 | 42441 |
| 56-MWZ-2 | 2437 | 207 | 178 | 2397 | 456 | 37 | 1106 | 48386 |
| 57-ZYX-1 | 4877 | 295 | 193 | 3350 | 768 | 27 | 1294 | 70561 |
| 58-ZYX | 2975 | 172 | 119 | 2680 | 517 | 8 | 1114 | 82395 |
| 59-824687-1 | 3378 | 326 | 135 | 3692 | 579 | 14 | 1537 | 69503 |
| 60-824687-2 | 2674 | 219 | 180 | 2785 | 487 | 16 | 1044 | 51292 |
| 61-825333-1 | 2076 | 157 | 204 | 2446 | 580 | 34 | 1170 | 51519 |
| 62-825333-2 | 2582 | 333 | 201 | 2677 | 500 | 25 | 1109 | 58147 |
| 63-828968-1 | 3491 | 209 | 176 | 3565 | 829 | 15 | 1806 | 64499 |
| 64-828968-2 | 2318 | 208 | 79 | 2428 | 433 | 13 | 998 | 84746 |
| 65-829197-1 | 4478 | 159 | 209 | 5108 | 1442 | 28 | 3626 | 80188 |
| 66-829197-2 | 3601 | 234 | 150 | 3403 | 701 | 19 | 1281 | 72825 |
| 67-837251-1 | 7248 | 333 | 222 | 6893 | 1823 | 34 | 3218 | 63557 |
| 68-837251-2 | 3905 | 248 | 214 | 3529 | 953 | 30 | 1903 | 51907 |
| 69-13502-1 | 2644 | 226 | 196 | 2434 | 500 | 20 | 989 | 48356 |
| 70-13502-2 | 2473 | 161 | 199 | 2473 | 570 | 24 | 1233 | 55947 |
